# Eukfinder: a pipeline to retrieve microbial eukaryote genome sequences from metagenomic data

**DOI:** 10.1128/mbio.00699-25

**Published:** 2025-04-10

**Authors:** Dandan Zhao, Dayana E. Salas-Leiva, Shelby K. Williams, Katherine A. Dunn, Jason D. Shao, Andrew J. Roger

**Affiliations:** 1 Institute for Comparative Genomics, Dalhousie University 3688, Halifax, Nova Scotia, Canada; 2 Department of Biochemistry and Molecular Biology, Dalhousie University 120880 , Halifax, Nova Scotia, Canada; 3 Department of Biochemistry, Cambridge University 98526, Cambridge, England, United Kingdom; University of Wisconsin-Madison, Madison, Wisconsin, USA

**Keywords:** eukaryotic microbial genomes, metagenomics, whole-genome shotgun sequencing

## Abstract

Whole-genome shotgun (WGS) metagenomic sequencing of microbial communities enables the discovery of the functions, physiologies, and evolutionary histories of prokaryotic and eukaryotic microbes. However, metagenomic studies of microbial eukaryotes lag due to challenges in identifying and assembling high-quality genomes from WGS data. To address this problem, we developed Eukfinder, a bioinformatics pipeline that identifies potential eukaryotic sequences from WGS metagenomic data, with a complementary binning workflow for recovering nuclear and mitochondrial genomes. Eukfinder uses two specialized databases for read/contig classification, customizable to specific data sets or environments. We tested Eukfinder on simulated gut microbiome data sets which included varying numbers of reads from the protist *Blastocystis*, a human gut commensal. We also applied Eukfinder to previously published human gut microbiome WGS metagenomic data to recover new genomes of *Blastocystis*. Compared to other workflows, Eukfinder offers the potential to recover high-quality, near-complete genomes of diverse eukaryotes, including different *Blastocystis* subtypes, without relying on a reference genome. With sufficient sequencing depth, Eukfinder outperforms similar tools for recovering eukaryotic genomes from metagenomic data. Eukfinder is a valuable tool for reference-independent and cultivation-free studies of eukaryotic microbial genomes from environmental WGS metagenomic samples.

**IMPORTANCE:**

Advancements in next-generation sequencing have made whole-genome shotgun (WGS) metagenomic sequencing an efficient method for *de novo* reconstruction of microbial genomes from various environments. Thousands of new prokaryotic genomes have been characterized; however, the large size and complexity of protistan genomes have hindered the use of WGS metagenomics to sample microbial eukaryotic diversity. Eukfinder enables the recovery of eukaryotic microbial genomes from environmental WGS metagenomic samples. Retrieval of high-quality protistan genomes from diverse metagenomic samples increases the number of reference genomes available. This aids future metagenomic investigations into the functions, physiologies, and evolutionary histories of eukaryotic microbes in the gut microbiome and other ecosystems.

## INTRODUCTION

Microbial eukaryotes are ubiquitous and inhabit every global ecosystem. Their genomes reveal physiological capacities, evolutionary histories, as well as their interactions with other microbes, their hosts, and their environment. Unfortunately, due to a lack of published complete genome sequences, high-throughput analyses of the population and evolutionary genomics of microbial eukaryotes have lagged behind those of prokaryotes. The availability of prokaryotic genome data has dramatically increased in recent years thanks in part to the use of metagenomic assembly and binning tools which allow for the study of uncultured microbial lineages (1, 2). Since the first application of a metagenomic sequencing approach to reconstruct near-complete bacterial genomes (3), hundreds of thousands of high-quality complete or draft metagenome-assembled genomes (MAGs) for bacterial and archaeal species have been constructed (4, 5). By contrast, the complexity of eukaryotic genomes (e.g., larger size, increased sequence complexity, and lower abundance in metagenomic data) has hindered the development of effective bioinformatic tools for generating eukaryotic MAGs (6). This is mainly because typical metagenomics tools are either prokaryote-centered or too cumbersome to use, especially for researchers who lack bioinformatic knowledge (7).

Eukaryotic genome reconstruction from metagenomic data has seen limited exploration. For instance, Beghini et al. applied a reference-genome-based read mapping and assembly method (hereafter referred to as “Refmapping”) to reconstruct 43 draft *Blastocystis* genomes from 2,154 gut metagenomic data sets, with completeness estimates ranging from 33% to 85% (8). West et al. developed EukRep, a machine learning k-mer-based tool for separating eukaryotic and prokaryotic sequences (9). Applying EukRep to 1,198 metagenomic data sets recovered 14 novel eukaryotic genomes with a median completeness of 91% (10). Tiara, a deep learning approach, identifies eukaryotic and organellar genomes within metagenomic data sets (11). Recent tools like EukHeist (12) and VEBA (13) utilize EukRep and Tiara, respectively. These studies demonstrate the feasibility of reconstructing microbial eukaryotic genomes from metagenomic data.

However, previous pipelines have limitations. Refmapping, for example, requires closely related reference genomes. Machine-learning approaches like EukRep and Tiara rely on models trained on reference genome sets, which can be a strength for detecting distantly related sequences when balanced and diverse training sets are available. However, the construction of custom databases for specific environments requires model retraining that can be time-intensive and challenging for underrepresented taxa. EukRep only allows for two classification states (prokaryotic or eukaryotic) which can result in misclassifying prokaryotic sequences as eukaryotic, leading to potential contamination of eukaryotic genome assemblies (11). In addition, Tiara and EukRep are designed to use contigs >3,000 bp and cannot accommodate shorter sequences.

To surmount these limitations, we developed Eukfinder, a bioinformatic tool designed to detect potential eukaryotic sequences from environmental metagenomes, supported by a binning workflow for recovering nuclear and mitochondrial genomes from these classifications. Eukfinder improves upon existing pipelines by allowing for the use of short reads and the ability to customize the search database. Specialized databases can be built by users to include reference genomes from representative organisms in the environment of interest. To demonstrate its utility, Eukfinder was applied to mock community data sets that included varying amounts of *Blastocystis* sequence reads as well as to real human gut metagenomic data sets. We examined the ability of Eukfinder to identify eukaryotic sequences focusing on *Blastocystis* genomes as a test case.

*Blastocystis* spp. (referred to as *Blastocystis* hereafter) are among the most prevalent microbial eukaryotes colonizing the GI tracts of different animals and humans; while they were once considered parasitic, they are now thought to be commensal (14, 15). Out of 17 currently known subtypes (STs), only 8 have published genomes (Table S1, as of March 2023) (16). Among these genomes, three (ST1, ST4, and ST7) are nearly complete (17–19), whereas the remainder are draft assemblies (20). Our analyses of gut metagenome data show that Eukfinder facilitates the reconstruction of microbial eukaryote genomes such as those of *Blastocystis*, recovering more complete nuclear and mitochondrial genomes than both reference mapping and machine learning-based methods. We also evaluate Eukfinder’s ability to handle a more complex data set from the Tara Oceans project. In all the foregoing analyses, we benchmark Eukfinder’s performance against competing approaches. We demonstrate that Eukfinder is a useful tool for the recovery of eukaryotic sequences from metagenomic data, aiding future studies into the functions, physiology, and evolutionary history of microbial eukaryotes.

## RESULTS

### Overview of the Eukfinder pipeline and the binning workflow

**Fig 1:**
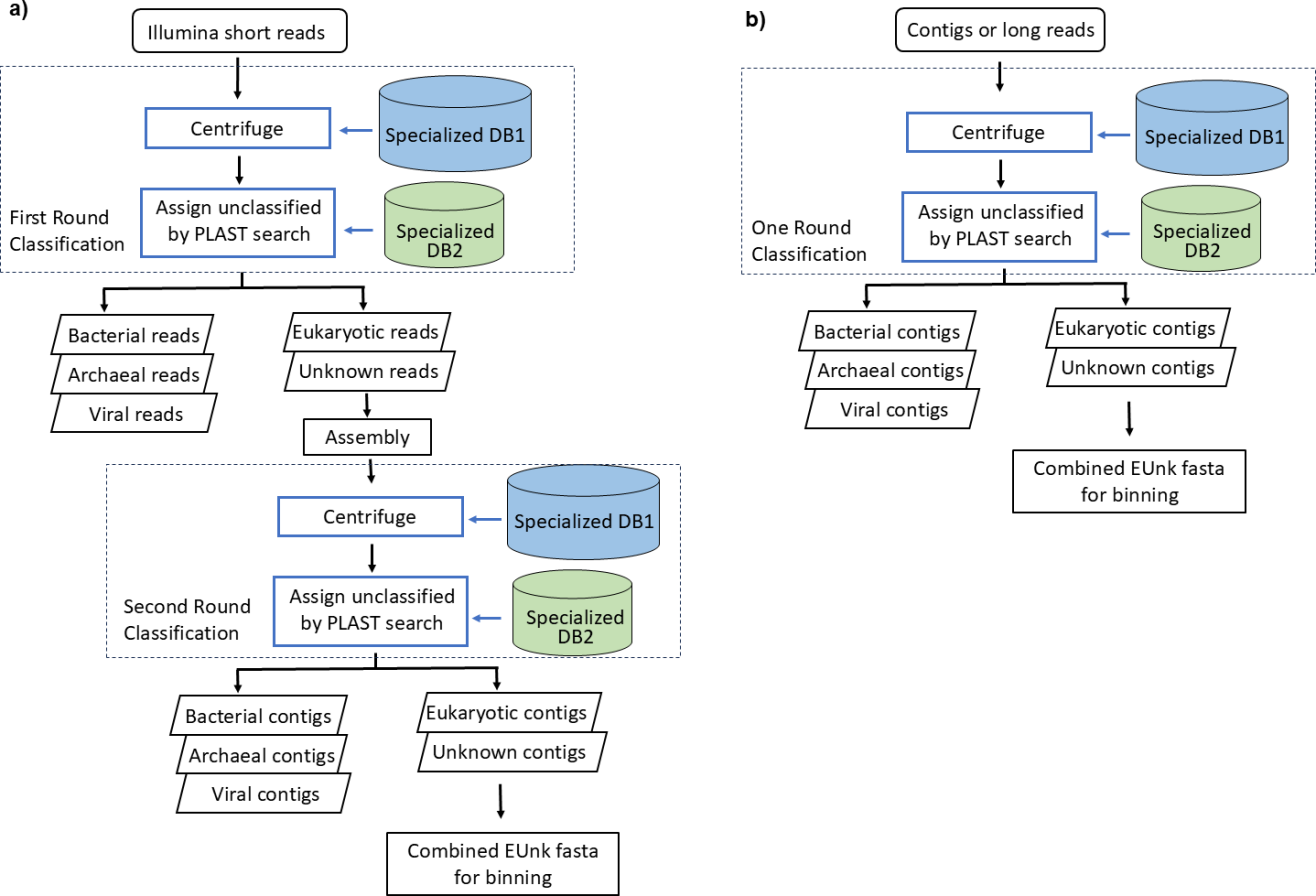
Schematic representation of Eukfinder workflows. Eukfinder is a taxonomic classification-based bioinformatics approach to retrieve microbial eukaryotic nuclear and mitochondrial genomes from WGS metagenomic sequencing data. Eukfinder has two different workflows based on the input files. (a) Eukfinder_short utilizes Illumina short reads, and the first round of classification assigns reads into 1 of 5 distinct taxonomic categories (Archaeal, Bacterial, Viral, Eukaryotic, and Unknown). Next, Eukaryotic and Unknown reads are assembled into contigs which undergo a second round of classification to generate potential eukaryotic sequences. (b) Eukfinder_long uses assembled contigs or long-read sequencing data and only performs one round of classification to select Eukaryotic and Unknown contigs. The potential eukaryotic contigs can then be further separated into MAGs by a separate binning workflow to generate draft eukaryotic nuclear and mitochondrial genomes.

Eukfinder is designed to retrieve eukaryotic genome sequences from metagenomic data (Fig. 1). It can process short Illumina metagenomic reads (processed with Eukfinder_short), assembled contiguous sequences (hereafter referred to as contigs), or metagenomic reads derived from long-read sequencing platforms, such as Oxford Nanopore or PacBio (processed with Eukfinder_long). Eukfinder_short first classifies short reads into five taxonomic categories (Archaeal, Bacterial, Viral, Eukaryotic, and Unknown) using Centrifuge (21), with a custom database (DB1), and PLAST (22), with a specialized database (DB2) (Fig. 1a) (details about database preparation are given in Supplemental Material). Reads classified as “Eukaryotic” or “Unknown” are assembled into contigs using metaSpades (23). These contigs are then reclassified with Centrifuge (DB1) and PLAST (DB2), and contigs assigned as “Eukaryotic” or “Unknown” are combined and treated as potential eukaryotic sequences. This output file from the Eukfinder pipeline is then processed using a binning workflow in which the binned sequences are classified based on depth of coverage and taxonomy, resulting in high-quality draft assemblies of nuclear and mitochondrial eukaryotic sequences. Eukfinder_long, used for contigs or long-read sequences, follows a similar workflow but includes only one classification round (Fig. 1b). We note that the binning workflow used here is separate from the main Eukfinder pipeline so that users may process the “Eukaryotic” or “Unknown” output file using whatever binning strategy they prefer. Full details and parameters for the current pipeline and workflow are given in Materials and Methods.

To test Eukfinder’s ability to retrieve *Blastocystis* genomes and examine its performance relative to other eukaryote metagenomic read classifiers, we benchmarked Eukfinder_short and Eukfinder_long along with three other methods on simulated mock community data sets. These data sets consist of synthetic human gut bacterial data (16.6M reads) and varying amounts of randomly selected *Blastocystis* ST1 Illumina reads (300K to 10M reads), creating eight mock metagenomic data sets in quadruplicate (Table S2; Fig. S1). To ensure that the results from competing methods were comparable, the same binning workflow was applied to all methods tested.

### Mock community data set analyses

**Fig 2:**
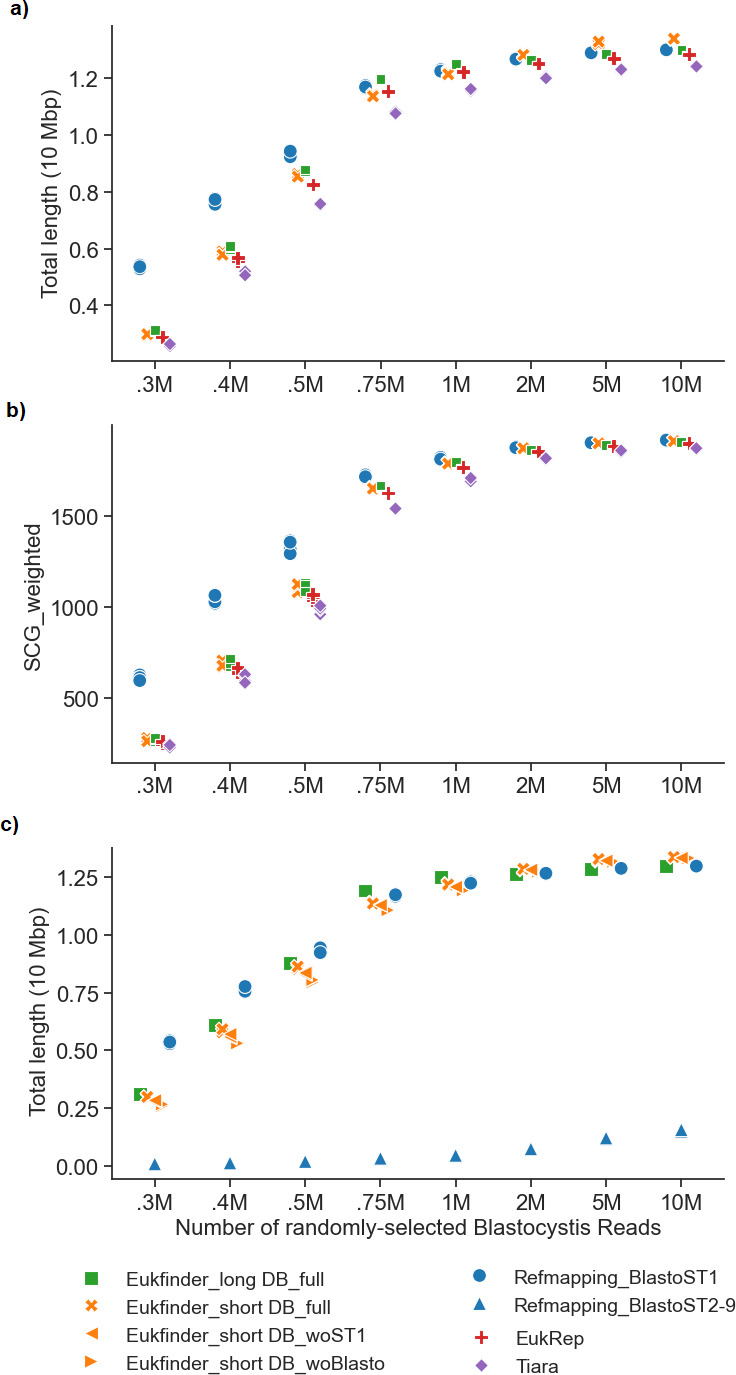
Comparison of methods (Eukfinder_short, Refmapping, Eukfinder_long, EukRep, and Tiara) to recover *Blastocystis* ST1 genomes in eight datasets consisting of bacterial mock metagenomic data (mix-51) with varying amounts of randomly selected *Blastocystis* ST1 Illumina sequencing reads. (a) The plot of the total length of *Blastocystis* ST1 contigs recovered by each method. (b) Plot of weighted single copy genes (SCG) (number of SCGs detected corrected by gene completion). (c) The plot of recovered *Blastocystis* ST1 total length using reference data sets excluding ST1. Eukfinder_short was reassessed with databases excluding the ST1 genome, but including other *Blastocystis* genomes, (woST1), and with all *Blastocystis* genomes excluded (woBlasto). Refmapping was reassessed using genomes from ST2-9 as the reference (Table S1).

We compared the Eukfinder short and long-read workflows with Refmapping (8), EukRep (9), and Tiara (11) to recover *Blastocystis* ST1 genomes from the eight groups of mock metagenomic data sets. Due to differences in input data for the methods examined, Eukfinder_short and Refmapping were used to examine short Illumina reads, while Eukfinder_long, EukRep, and Tiara were used to examine assembled contigs of these reads. We assessed genome completeness using QUAST (24), measuring total genome length, genome fraction, misassembled length, NG50, and LG50 (Fig. 2; Fig. S2; File S1). In addition, we used a set of 19,43 manually selected single-copy genes (SCGs) for a more comprehensive completeness assessment (Fig. 2b) compared to BUSCO’s limited SCG sets (25, 26).

Eukfinder_long, EukRep, and Tiara were compared based on the total length and genome completion of *Blastocystis* ST1 genomes with contigs ≥1,000 bp. Eukfinder_long produced significantly longer and more complete genomes than EukRep (0.8%–3.4%) and Tiara (2.9%–8%) (Bonferroni corrected *P* < 0.05; Fig. S2; Tables S3 and S4; File S1). Tiara had the shortest and least complete genomes among the three methods (Fig. 2a and b; Fig. S2; File S1). QUAST analysis revealed that Eukfinder_long had the lowest misassembled lengths and percentages across all data sets, reflecting good assembly accuracy (Fig. S2c and d; File S1). NG50 and LG50 metrics further supported Eukfinder_long’s performance. Eukfinder_long achieved significantly higher NG50 values and lower LG50 metrics compared to EukRep and Tiara, indicating more contiguous assemblies with fewer and longer contigs across all read counts (Fig. S2e and f; File S1).

For short-read comparisons, Refmapping (local mode) recovered the most complete genomes at lower read counts (≤1M) compared to Eukfinder_short (Fig. 2a; Fig. S3; File S1). Refmapping_local outperformed Eukfinder_short by 10%–18% at 300K–500K reads. This gap narrowed at higher read counts, with Refmapping_local only 1%–3.5% better at 750 K-1M reads. Beyond 1M reads, Eukfinder_short recovered significantly longer and more complete genomes than Refmapping (*P* < 0.005; Table S5). Refmapping_local showed higher misassembly rates, particularly at lower read counts (Fig. S2c and d; File S1), which might account for the longer total length. We also investigated Refmapping’s global mode (end-to-end alignment) performance, as it is frequently employed for genome recovery. Refmapping_global alignment showed less genome completion compared to local mode (Fig. S3; File S1), especially at lower read counts. With 500K reads, global alignment achieved <20% genome completion, compared to >60% with local alignment. Performance improved with more reads but remained inferior to local mode at 1M reads (60% vs. 90% completeness). At >2M reads, Refmapping_global’s performance approached that of other methods, achieving >85% completeness.

### Performance in the absence of reference genomes in the databases

Reference mapping methods rely heavily on the quality and availability of draft reference genomes for organisms closely related to the eukaryotes in the metagenomic data. Similarly, Eukfinder benefits if these reference genomes are included in its Centrifuge and PLAST databases. To assess the impact of reference genomes, we replaced the *Blastocystis* ST1 genome with genomes from *Blastocystis* ST2-ST9 (ST2-9) for Refmapping and created two modified databases for Eukfinder: one excluding ST1 (DB_woST1) and one excluding all *Blastocystis* genomes (DB_woBlasto).

Refmapping with ST2-9 performed poorly, recovering less than 12% of the *Blastocystis* ST1 genome and producing shorter genomes regardless of read count (Fig. 2c; Fig. S3a; File S1). By contrast, Eukfinder_short maintained similar genome lengths, with only a <2% reduction using DB_woST1 and <4.3% with DB_woBlasto (Fig. 2c; Fig. S3; File S1). The reduction in genome completeness without ST1 was minimal for Eukfinder_short (<1% to <4.5%) compared to Refmapping (36%–88%) (Fig. 2c; Fig. S3; File S1).

QUAST analysis of misassembled contig length (Fig. S3c; File S1) and percentage of misassembled contigs (Fig. S3d; File S1) showed Eukfinder_short had the lowest misassembled lengths and percentages, indicating high assembly accuracy even without ST1. Refmapping, using ST2-9, exhibited higher misassembled lengths and percentages, reflecting its dependence on a close reference genome. These findings highlight Eukfinder’s robustness in producing accurate and reliable assemblies, even without specific reference genomes.

Using the DB_woST1 and DB_woBlasto databases, the Eukfinder_long workflow recovered *Blastocystis* ST1 genomes of similar size to the ST1 genomes recovered using the full databases. Closer inspection showed that Eukfinder classified the *Blastocystis* contigs as “Unknown,” which were included in the subsequent binning step (Fig. S4). This explains how Eukfinder was able to recover similarly sized genomes both with and without closely related genomes included in its databases.

### Precision and recall analyses

**Fig 3:**
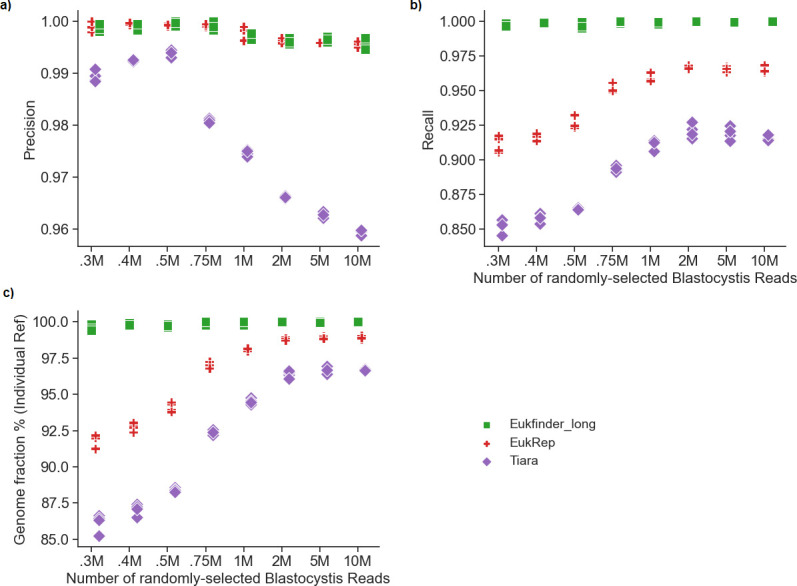
Precision and recall of the genomes recovered by Eukfinder_long, EukRep, and Tiara. (a) Average precision, measured as the fraction of true *Blastocystis* reads among all reads assigned as *Blastocystis*, and (b) average recall, measured as the fraction of *Blastocystis* reads that were recovered, at each read depth for the three long-read methods (Eukfinder_long, EukRep, and Tiara). Precision = true positives/(true positives + false positives). Recall = true positives/(true positives + false negatives). (c) The fraction of *Blastocystis* recovered from each mock community data set was assessed using QUAST using the true *Blastocystis* contigs (Individual Ref, based on input reads rather than complete reference genome) as the reference.

We examined the precision and recall of genomes recovered by the five methods (Fig. S5; see Materials and Methods for details). Precision measures the proportion of correctly identified *Blastocystis* contigs (true positives divided by true positives plus false positives), while recall measures the proportion of all *Blastocystis* contigs correctly identified (true positives divided by true positives plus false negatives). Both Eukfinder_long and EukRep had high precision (~100%; Fig. 3; File S2). Tiara’s precision ranged from 96% to 99%, depending on the number of *Blastocystis* reads. High precision indicates accurate identification of *Blastocystis* contigs. Recall, however, varied more significantly. Eukfinder_long had nearly perfect recall (>99.7%) in all cases, while EukRep’s recall ranged from 91-97%, improving with more reads (Fig. 3a). Tiara had the lowest recall (85-92%; Fig. 3a), missing up to 15% of contigs in some cases (Fig. 3b).

We also used QUAST with true *Blastocystis* contigs from each mock community data set (based on read number, termed “individual Ref”) as the reference to assess completeness by each method (Fig. 3c; Fig. S5). Eukfinder_long and _short consistently recovered the largest fraction of the individual Ref, while Refmapping performed poorly with <0.75M reads. Refmapping showed the highest completeness with the complete ST1 genome but the lowest with individual Ref. This discrepancy likely stems from the use of local alignment mode, which retains partially aligned reads, including non-target sequences or misassembled regions, reducing overall completeness and accuracy.

### Real human gut metagenomic samples

**Fig 4:**
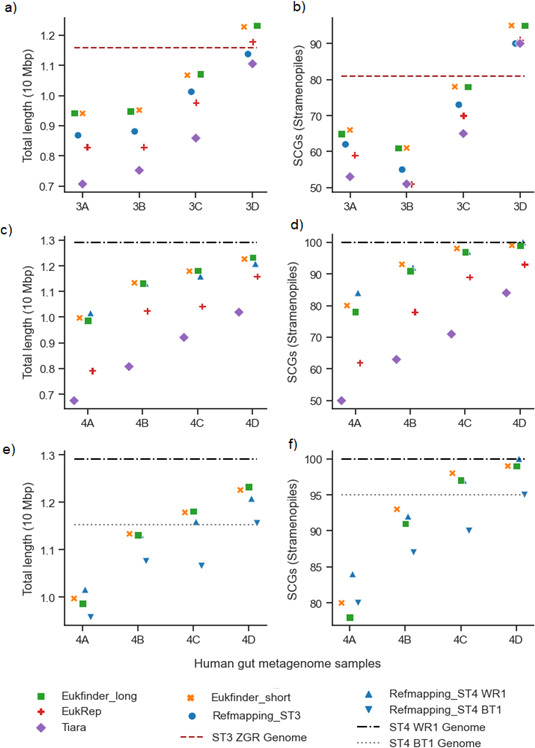
*Blastocystis* ST3 and ST4 genomes recovered from eight human gut metagenome samples (3A–D and 4A–D, Table S6) using Refmapping, Eukfinder_short, Eukfinder_long, EukRep, and Tiara. Genome completeness in ST3 containing samples (Refmapping reference genome ST3 ZGR) based on (a) total length (10 Mbp) and (b) number of SCGs detected using BUSCO (stramenophile_odb10). Genome completeness in ST4 containing samples (Refmapping reference genome ST4 WR1) based on (c) total length (10 Mbp) and (d) number of SCGs detected using BUSCO (stramenophile_odb10). Reanalysis of Refmapping ST4 genome completeness using reference genome ST4 BT1, (e) total length (10 Mbp), and (f) number of SCGs detected using BUSCO (stramenophile_odb10). Tiara was run with a kmer of 4 and a probability threshold of 0.7, and Refmapping was run in local mode. Lines representing reference genome sizes are shown for *Blastocystis* ST3 ZGR (dash), ST4 WR1 (dash-dot), and ST4 BT1 (dotted) for comparison.

Eight human gut metagenomic samples containing *Blastocystis* reads (Table S6) were examined using Eukfinder (short and long), Refmapping, EukRep, and Tiara to assess genome recovery. Four samples contained *Blastocystis* ST3 sequences (3A–3D) and four contained ST4 sequences (4A–4D). Total genome length, genome fraction, NG50, %GC, and single-copy genes (SCG) were assessed using QUAST and BUSCO (stramenophile_odb10) (Fig. 4; Fig. S6 to S9; File S3).

Eukfinder consistently recovered longer and more complete genomes compared to other methods. Eukfinder_short recovered significantly longer genome lengths than Refmapping (*P* = 0.013) in most samples, with no significant differences in genome fraction and SCGs (*P* = 0.20, 0.06, respectively) (Fig. 4; File S3). Eukfinder_long outperformed EukRep and Tiara in total genome length (vs. EukRep *P* = 7.6e−5; vs. Tiara *P* = 6.2e−5), genome fraction (vs. EukRep *P* = 1.6e−4; vs. Tiara *P* = 5.2e−5), and SCG count (vs. EukRep *P* = 2.0e−4; vs. Tiara *P* = 7.1e−4) (Fig. 4a through d; File S3). Eukfinder_long recovered 2%–15% more complete genomes than EukRep and 6%–20% more than Tiara (Fig. 4a and c; File S3).

For ST3 samples, short read methods recovered genomes between 8.7 and 12.3 Mbp with 74-97% completion, while long read methods recovered genomes between 7.5 and 12.3 Mbp with 59%–97% completion (Fig. 4a and b; Fig. S6a; File S3). Sample 3D had genomes longer than the published reference genome, with Eukfinder_long, Eukfinder_short, and EukRep recovering 12.315 Mb, 12.270 Mb, and 11.783 Mb, respectively (Fig. 4a; File S3). All methods recovered more SCGs in sample 3D than the published reference genome (Fig. 4b; Fig. S6b and S7), which had been estimated as incomplete (20).

For ST4 samples, short read methods recovered genomes between 10 and 12.3 Mbp with 76%–94% completion, while long read methods recovered genomes between 7.6 and 12.3 Mbp with 56%–94% completion (Fig. 4c and d; Fig. S8 and S9; File S3). Refmapping with the most *Blastocystis* reads (sample 4D, 2.63M reads) matched the number of SCGs in the complete ST4 WR1 reference genome (Table S1; Fig. 4e). Using a smaller, incomplete ST4 genome (BT1) as the reference for Refmapping resulted in shorter genomes and fewer SCGs across all ST4 samples (Fig. 4e and f; Fig. S10; File S3), highlighting Refmapping’s dependency on reference quality.

Eukfinder also demonstrated the ability to recover multiple eukaryotic genomes from a single data set. In sample 4D (Table S7), Eukfinder also recovered *Dientamoeba* sequences from the data. While EukRep and Tiara also recovered *Dientamoeba* in this sample, Eukfinder_short recovered the largest set of *Dientamoeba* contigs (5.35 Mbp, 2,190 contigs). BUSCO analysis (eukaryota_odb10) showed 2.0% completeness for Eukfinder_short, Eukfinder_long, and EukRep, while Tiara achieved 0.4%. The absence of a reference genome for *Dientamoeba* meant Refmapping could not be used. Although the recovered *Dientamoeba* genome was very incomplete, these findings underscore Eukfinder’s ability to recover multiple eukaryotic genomes from a single data set.

Additional eukaryotic genome sequences were also recovered in other human samples including *Cryptosproridium* in sample 3A, *Entamoeba* in sample 3C, and *Saccharomyces cerevisiae* in samples 3C, 4A, 4B, and 4C. Based on the convergence of several methods, 10 contigs of the *Saccharomyces cerevisiae* (Fig. S11) were identified in sample 4C. Of these contigs, Eukfinder_short classified 8 (16,425 bp), Eukfinder_long and EukRep classified 6 (14,993 and 15,283 bp, respectively), and Tiara classified five as eukaryotic (12,389 bp) and two as unknown (2,834 bp). Although these contigs were too few to form a bin during binning, their identification highlights Eukfinder’s capability to identify eukaryotic sequences, even in cases of low abundance or fragmented genomic representation.

### Tara Oceans data set

To assess the scalability of our approach with more complex metagenomic data sets, we analyzed a Mediterranean Sea site (SRA accession number: ERR868402) from the Tara Oceans study (27). After assembly with metaSPAdes (details in Supplemental Material), 64,535 contigs (≥1,000 bp) were obtained. Using multiple methods (see Supplemental Material), we determined, to the best of our ability, the origin of each contig to evaluate the performance of the methods (Eukfinder_long, EukRep, and Tiara). The classification was deemed unknown or unclear for 11,124 contigs which were excluded. The remaining 53,411 contigs (3,733 Eukaryota, 49,678 Prokaryota or Virus) were then analyzed using Eukfinder_long, EukRep, and Tiara, and their performances were assessed (Table S8). For comparability, we used the same databases for Eukfinder as for all previous analyses. Because these databases were built for gut metagenome analyses, we tested Eukfinder both with the standard settings above (“strict”) and with more “lenient” settings that allowed for lower percentage identity, less coverage, and shorter hit lengths (Table S9). Eukfinder_long “strict” considering reads classified as Eukaryote (Euk) only had the highest precision (0.77) but the lowest recall (0.25), whereas Eukfinder_long “strict” considering reads classified as Eukaryote and Unknown (EUnk) had the lowest precision (0.1) but the highest recall (0.95) (Table S8). With more lenient parameter settings, Eukfinder_long appeared to better balance precision (0.4) and recall (0.52) (Table S8). The performance of EukRep, Tiara (Euk), and Tiara (EUnk) ranged from 0.3 to 0.36 (precision) and 0.48–0.5 (recall).

### Resource usage and computational efficiency

We compared the performance of Eukfinder against Refmapping, EukRep, and Tiara in terms of computational efficiency. All computations were performed on a Lenovo SD350 server with 40 Intel “Skylake” cores clocked at 2.4 GHz and having 188 GB RAM. For each method, we allocated 10 CPUs, except for EukRep, which uses a single CPU. Benchmark tests were conducted on a data set from Human sample 3A, consisting of 30.2 million reads and 6.0 Gbp of total data.

The results, summarized in Table S10, showed runtimes for each method. Eukfinder_short, which uses two rounds of classification, took 557 minutes, while Eukfinder_long, which uses one round, took 98 minutes, closer in runtime to EukRep (85 minutes) and Tiara (82 minutes). Refmapping was the fastest, completed in 11 minutes.

The process of assembling all reads using metaSPAdes required 81 minutes and 20 seconds, whereas focusing only on the reads identified as “Eukaryotic” or “Unknown” after Eukfinder_short’s first classification round reduced the assembling time to 11 minutes and 30 seconds. This approach lessens the computational strain by trimming down the data set before assembly.

In addition, we observed that Eukfinder’s performance in terms of assembly quality and computational efficiency highlights its practical utility in handling large-scale metagenomic studies. Eukfinder_short’s classification steps, though time-intensive, contributed to more complete genomes with marginally longer contigs, especially in complex data sets with large numbers of reads. Eukfinder_long maintained a balance between speed and performance, making it a viable option for large-scale metagenomic studies where computational resources are a consideration.

### Recovering MRO genomes

**Fig 5:**
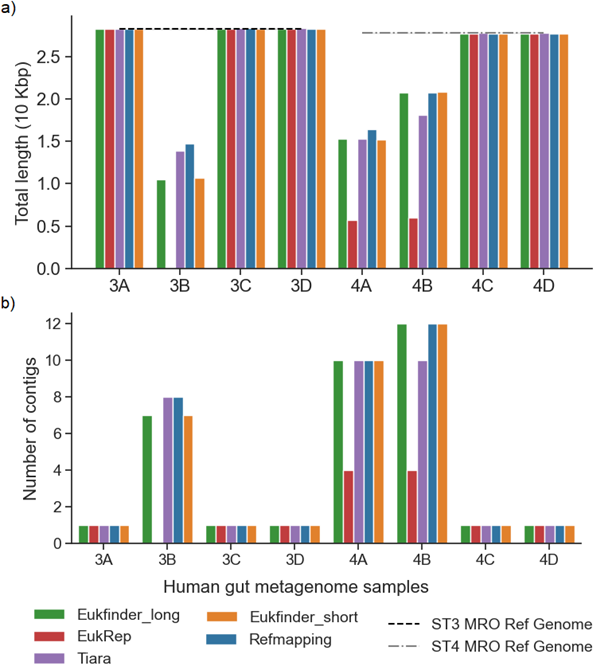
Comparison of the methods (Eukfinder_long, EukRep, Tiara, Eukfinder_short, and Refmapping) to recover *Blastocystis* mitochondrial related organellar (MRO) genomes from the eight human gut metagenome data sets, (a) Total length of MRO genomes (10 kbp); (b) number of contigs recovered. The MRO reference genome size for ST3 (dashed line) and ST4 (dashed-dotted line) is shown.

We assessed whether reads recovered with Eukfinder and other methods could also recover mitochondrial genomes from metagenomic data sets using the additional binning workflow. We used the eight human gut metagenomic data sets to attempt recovery of *Blastocystis* mitochondrion-related organelle (MRO) genomes. In five samples (3A, 3C, 3D, 4C, and 4D), all methods recovered single complete MRO genomes (Fig. 5). No or partial MRO genomes were recovered in the remaining three samples (3B, 4A, and 4B). EukRep failed to recover an MRO genome for sample 3B, and the MRO genomes from 4A and 4B were smaller than those recovered by other methods (Fig. 5).

## DISCUSSION

Here we introduced Eukfinder, a novel bioinformatics pipeline for retrieving eukaryotic sequences from metagenomic data, with an additional workflow for recovering nuclear and mitochondrial genomes. We benchmarked Eukfinder against Refmapping, EukRep, and Tiara using simulated and real metagenomic data sets and found that Eukfinder consistently outperforms these tools in genome completeness, contiguity, and accuracy, especially with high read counts. Eukfinder has several key advantages: (i) it can utilize both short reads and contigs as input, (ii) it is less dependent than competing tools on the availability of reference sequences, and (iii) its databases can be straightforwardly customized to specific environments, maximizing genome recovery.

Previous studies investigating taxonomic and functional profiling of metagenome data found that although raw and assembled reads yielded similar results when metagenomes were very large, assembly methods performed better (28). Despite differences in input data (raw vs. assembled reads) and classification rounds (two vs. one), Eukfinder_short and Eukfinder_long produced similar results. The first round of Eukfinder_short serves as a filtering step, eliminating most prokaryotic sequences before assembly and decreasing assembly time. In our human metagenome samples, Eukfinder_short resulted in longer contigs and higher NG50.

Surprisingly, Eukfinder performs well even when a closely related reference genome is not included in the databases. This can be rationalized as follows. There are relatively few whole-genome sequences completed for microbial eukaryotes, whereas a vast diversity of prokaryotic genomes are complete and widely available. If the databases contain a large and diverse set of prokaryotic genomes, then the classification of bacterial or archaeal contigs will be accurate with few false negatives. The remaining contigs will then be classified as eukaryotic or unknown. If both the Eukaryotic and Unknown groups are used in the follow-up binning workflow as done here, this allows the identification of both known and potentially unknown eukaryotic genomes. Our analyses excluding *Blastocystis* from the database demonstrated that Eukfinder maintains high genome completeness and low misassembly rates in stark contrast to Refmapping, which exhibited a dramatic performance drop when closely related reference genomes were absent. One caveat to our analyses is that our benchmarking of Tiara was based on its default pipeline where only contigs classified as Eukaryotic were analyzed further in the binning step. Incorporating unknown contigs into the binning process, as outlined in Tiara’s extended pipeline (https://ibe-uw.github.io/tiara/eukaryotic_pipeline.html), is likely to improve its ability to recover eukaryotic genomes. We tested this for the TARA Oceans Mediterranean Sea data set and observed a small improvement in recall (i.e., the proportion of actual eukaryotic reads that were captured), although at the expense of precision (i.e., a higher false-positive rate: Fig. S8). Analyses of a much wider set of metagenomic data sets using both Eukaryotic and Unknown bins for both Eukfinder and Tiara are needed to provide a more thorough comparison of their performances.

Eukfinder is also able to recover sequences from multiple eukaryotic genomes from complex data sets. In our analyses of gut metagenomes, in addition to *Blastocystis*, Eukfinder recovered sequences from other gut protists in various samples including *Dientamoeba*, *Cryptosporidium,* and *Entamoeba*, as well as fungi such as *Saccharomyces cerevisiae*. In one sample (4D), Eukfinder assembled ~5 Mb of a *Dientamoeba* genome at the same time as recovering a near-complete *Blastocystis* ST4 genome (Fig. 4). This illustrates that Eukfinder and our binning workflow are generally useful tools even in environments with multiple eukaryotic species.

Our analyses of the Tara Oceans Mediterranean Sea data set as a test for Euk_finder, EukRep, and Tiara indicate that the complexity of this data set poses challenges for all of these methods. Most notable is the tradeoff between precision and recall. Recall here measures the proportion of actual eukaryotic reads that are captured and is an especially important metric when the goal is to capture all potential eukaryotic reads. Eukfinder with default “strict” parameters using the EUnk output file captures 95% of the real eukaryotic reads, but at the expense of a high false-positive rate—90% of the positives are false. However, if the goal is to maximize the completeness of recovered eukaryotic genomes, high recall with a high false-positive rate may not be a problem because putative positives can be further scrutinized in downstream binning workflows using read coverage depth, identified rRNA sequences, and taxonomic identities of contigs within bins. Alternatively, if a better balance between precision and recall is desired, then Eukfinder under the “lenient” parameter settings and restricting attention to only the Euk output file achieves this and performs slightly better than either EukRep, Tiara (Euk), or Tiara (Eunk). It is somewhat surprising that Eukfinder, with its default databases optimized for gut metagenome analysis, performs relatively well (in comparison with competing methods) on the Mediterranean Sea data set; this may be due to the fact that the core functionality in ocean and gut microbiomes substantially overlap (29). Nevertheless, customizing Eukfinder’s databases to include more genome data from the marine microbial ecosystems will likely improve its performance even further and is recommended for users wanting to pursue this further.

Eukfinder’s classification approach using Centrifuge and PLAST is important for its performance, balancing sensitivity and specificity in read classification, leading to more accurate genome assemblies. Future work could integrate additional algorithms or machine learning techniques to further enhance performance. Eukfinder offers user-friendly features and customizable databases, making it accessible to researchers with varying levels of bioinformatics expertise. Building specialized databases tailored to specific environments or research questions enhances their applicability across diverse metagenomic studies. Future research could expand its application to other ecosystems, such as marine or soil environments, to uncover new microbial diversity insights.

Beyond identifying eukaryotic sequences in environmental metagenomic data, Eukfinder can tackle other genomics-related problems, such as decontaminating eukaryotic genome sequence data when sequences are obtained from DNA extracted from cultures with prokaryotes present. This approach aids in *de novo* genome assembly of understudied and poorly understood eukaryotic taxa. Eukfinder can also pre-screen environmental sequences for potential eukaryotic community members, informing decisions during data collection, such as re-sequencing or re-sampling. Eukfinder works best with genome sizes <20M and large numbers of eukaryotic reads. Given that eukaryotic reads generally make up a small proportion (typically <5%) of WGS data in many metagenomic samples, the number of sequencing reads needed to recover complete eukaryotic genomes using Eukfinder is often large. Our analyses of real human gut samples, however, suggest that it can be difficult to predict how many metagenomic reads are required to obtain near-complete eukaryotic genomes. For example, sample 4D had over 75M metagenomic reads, 3.5% of which came from *Blastocystis* ST4 (2.6M reads) and recovered a nearly complete genome. Yet, sample 3D had nearly 120M reads with just 0.68% being *Blastocystis* ST3 (0.82M reads 3× fewer) but recovered a more complete genome than the current reference genome for that subtype. Clearly, the complexity of the target genome in terms of size and repeat sequence content, the identity of the associated prokaryotes and their representation in the databases, and the relationship of reference eukaryotic genomes in the databases to the target genome will all be important factors influencing the number of reads required for its assembly.

With respect to the detailed classification of the metagenomic reads, Eukfinder plus the binning workflow we used provides an alternative to recent tools like EukHeist (12), VEBA (13), and Whokaryote (30), which build on EukRep and Tiara. Eukfinder benefits from its ability to use short raw reads and increased read recall compared to both EukRep and Tiara. While EukHeist specializes in eukaryotic genome recovery, Eukfinder extends beyond this by incorporating bacterial, archaeal, and viral genome retrieval capabilities, providing a holistic approach similar to VEBA’s modular and flexible framework. We note that although the binning workflow utilized in this paper for all methods uses MyCC (31), other binning tools like CONCOCT (32), MetaBAT2 (33), and MaxBin2 (34) could easily be used with Eukfinder’s output.

In conclusion, sequences identified by Eukfinder can be used to efficiently generate high-quality, near-complete nuclear and mitochondrial genomes from diverse microbial eukaryotes such as *Blastocystis*. Its ability to customize databases and employ sophisticated classification steps makes it highly adaptable to specific environmental contexts. We anticipate that Eukfinder will be a valuable tool for reference-independent and cultivation-free eukaryotic microbial genome recovery from environmental WGS metagenomic samples.

## MATERIALS AND METHODS

### Implementation of Eukfinder

Eukfinder is a taxonomy-classification-based pipeline for recovering microbial eukaryotic genome sequences from WGS metagenomic data sets (Fig. 1). The Eukfinder workflow uses two databases for classification: one compatible with Centrifuge (21) and one compatible with PLAST (22). The detailed database preparation is described in Supplemental methods.

#### Eukfinder pipeline for short sequences

Short reads require pre-processing to remove low-quality reads, sequencing adapters, and host reads before taxonomic classification with Centrifuge. Eukfinder’s “read_prep” handles this pre-processing using Trimmomatic v0.36 (35) to trim adapters, filter low-quality bases (<Q25), and remove short reads (<40 bp). Human host reads are removed by mapping to the host reference genome (GRCh38) using Bowtie2 v2.3.1 (36). Processed reads are then classified with Centrifuge v1.0.4, using DB1 with a minimum hit length of 40. Three result sequence files are produced: two for paired-end reads and one for unpaired reads.

Eukfinder_short uses these pre-processed reads and Centrifuge results to separate sequences by taxonomic classification (bacteria, archaea, eukaryote, virus, and unknown). Unknown sequences are further analyzed with PLAST (e-value = 0.01, identity = 70%, hit length coverage = 30%) against DB2. Reads classified as eukaryotes or unknown are combined and assembled with metaSPAdes-3.13.1 (23) using default parameters. Contigs ≥ 1,000 bp are then reclassified with Centrifuge (DB1) and PLAST (DB2). Eukaryote and unknown contigs (EUnk) undergo a binning process to recover eukaryotic genomes.

#### Eukfinder pipeline for long sequences

For long-read sequences (PacBio or Nanopore) or contigs, Eukfinder_long is used. This requires one round of taxonomic classification with Centrifuge (min hit length = 100) and PLAST (same parameters as Eukfinder_short). Contigs are separated into five categories (bacteria, archaea, eukaryotes, viruses, and unknown). Eukaryote and Unknown contigs (EUnk) are used in a binning workflow to recover eukaryotic genomes. All parameters used in this study are listed in Table S9.

### Binning workflow

Contigs identified as eukaryotic or unknown (EUnk) by the Eukfinder pipeline and eukaryotic by other methods were subjected to a binning workflow, which incorporated Centrifuge outputs, MyCC (31), Metaxa2 (37), and coverage depth to enhance contig classification and recover nuclear and mitochondrial genomes. Three separate MyCC analyses with different k-mers (4-mer, 5-mer, 5-6-mer) were used (Fig. S12). Read coverage depth for each assembly was calculated by mapping short reads to contigs with Bowtie2, sorted and indexed using SAMtools v1.9 (38), and generating depth files with MetaBat2 (33) (jgi_summarize_bam_contig_depths). LSU/SSU rRNA and mitochondrial sequences in the EUnk-assemblies were identified using Metaxa2 (align length >300, identity >90%). A nucleotide-based PLAST search was conducted using the contigs as queries against the NCBI-NT database (Jan 2019), with taxonomic IDs assigned using acc2tax v0.6 (github.com/richardmleggett). Database preparation for the binning workflow is detailed in Supplemental Material.

Read coverage depth, identified rRNA sequences, and taxonomic identities of contigs were mapped to corresponding MyCC bins. For a contig to be included in the final eukaryotic bin(s), the following criteria were applied:

- Depth of coverage could not exceed that of the SSU rRNA gene.
- The best PLAST hit could not be a prokaryote or virus with >90% identity over an aligned length ≥1,000 bp.
- Contigs identified as mitochondrial by Metaxa2 and PLAST were marked as mitochondrial genomes.
- Contigs identified as eukaryotic by Metaxa2, Centrifuge, and/or PLAST.
- Contigs appear at least twice in potential eukaryotic clusters across the three MyCC k-mers.

A schematic of supervised binning is shown in Fig. S13. This binning process was applied consistently across all methods (Eukfinder, EukRep, Tiara, and Refmapping) for comparative benchmarking. While we provide this binning workflow as a complementary step to the Eukfinder pipeline, it is not currently implemented in the Eukfinder pipeline.

### Mock community data sets

To test Eukfinder’s capabilities, we used simulated sequencing read files from a synthetic metagenome data set and randomly selected reads from the Illumina sequencing data set that generated the published genome of *Blastocystis* sp. ST1 (ATCC 50177/Nand II). The synthetic metagenome data set was the “Mix-51-staggered” human gut metagenome sequencing, downloaded from NCBI (SRA accession number: SRR8304765) (39). This data set was constructed from 51 human gut bacterial isolates, mixed in staggered molar amounts, with a total of 16.6M reads.

The Illumina sequencing reads that generated the published genome of *Blastocystis* sp. ST1 were mapped to the genome (GCA_001651215.1), and over 34 million reads mapping to *Blastocystis* sp. ST1 were extracted. To examine the impact of varying *Blastocystis* read counts on genome recovery, eight subsets of reads (ranging from 300K to 10M) were randomly selected from the 34 million Illumina *Blastocystis* reads and combined with all reads from the “Mix-51-staggered” data set. This process was repeated four times for each subset, resulting in 32 mock communities. The total numbers of Illumina ST1 reads, the fraction of ST1 reads, and the number of contigs ≥1,000 in each mock community are listed in Table S2.

### Benchmarking with mock communities

For fairness, we first identified the parameters for each program that maximized genome completeness and contiguity. The detailed methods employed to find the best parameters for EukRep (9), Tiara (11), and Refmapping (8) are described in Supplemental Material. Using the optimized parameters, we ran EukRep with lenient mode and default eukaryotic “tie” decision, Tiara with a k-mer of 4 and a probability threshold of 0.7, and Refmapping local mode for all data sets.

The published *Blastocystis* sp. ST1 ATCC 50177/Nand II genome (GCA_001651215.1) was constructed using both Illumina and 454 reads, making it unsuitable for direct benchmarking with our Illumina-only data sets. We assembled the 34M cleaned ST1 Illumina reads, generating 1549 contigs (>1,000 bp), referred to as the ST1 cleaned genome. This genome was used as the reference for Refmapping and Eukfinder_short workflows.

For Eukfinder_short, the combinations of randomly selected *Blastocystis* sp. ST1 reads and synthetic metagenome data sets were processed as outlined in Fig. 1a and Fig. S1. For Refmapping, metagenomic short reads were mapped to the ST1 cleaned genome using Bowtie2 in local mode, assembled with metaSPAdes-3.13.1, and contigs ≥1,000 bp were used in the binning workflow.

Randomly selected ST1 reads (300K to 10M) combined with the “Mix-51-staggered” synthetic metagenome were assembled with metaSPAdes-3.13.1, and contigs ≥ 1,000 bp were used as input for Eukfinder_long, EukRep, and Tiara (Fig. S1). Reads identified as eukaryotic (EukRep, Tiara) or EUnk (Eukfinder_long) were used in the binning workflow.

### Precision and recall

To assess precision and recall, reads from *Blastocystis* ST1 and “Mix-51-staggered” SRR8304765 were mapped onto contigs for each assembly using Bowtie2. Coverage depth was calculated using SAMtools v1.9, and depth files were generated with MetaBat2. Contigs without bacterial reads were extracted and classified using Centrifuge, Diamond (40), and PLAST.

Contigs with mapped ST1 reads classified as *Blastocystis* formed the individual reference genome (IndRef) for each of the 32 mock communities (Fig. S5). These IndRef genomes served as the ground truth for precision and recall analysis. Precision was measured as true positives divided by true positives plus false positives and recall as true positives divided by true positives plus false negatives. True positives were *Blastocystis* contigs correctly identified as such, and false positives were bacterial contigs misidentified as *Blastocystis*. True negatives were bacterial contigs identified correctly, and false negatives were *Blastocystis* contigs misidentified as bacterial. Genome fraction recovered by the methods was assessed using QUAST v5.0.2 (24) with IndRef contigs from each set of subsampled *Blastocystis* reads as the reference.

### Human gut shotgun metagenomic data

To test Eukfinder’s effectiveness, we applied it to eight human gut metagenome samples known to contain *Blastocystis* (Table S6). Raw sequencing data were downloaded from the NCBI Short Read Archive using the SRA toolkit. Quality control of the reads was performed using the Eukfinder preparation step to remove the adaptor, low-quality reads, and human host reads. These samples ranged from 8.8 to 23.3 Gbp in total size and 48.9M to 119.7M raw reads, with 415K to 2.6M reads classified as *Blastocystis* by Centrifuge (minimal hit length 30 bp). Eukfinder_short and Refmapping (using ST3 or ST4 reference genome) analyzed the short reads. Short reads were also assembled with metaSPAdes-3.13.1, and contigs ≥1,000 bp were input into Eukfinder_long, EukRep, and Tiara.

The ability of the methods to recover other eukaryotic signals was assessed by screening all human gut metagenome samples using Metaxa2 for eukaryotic signals. The eukaryotic signal identified was further confirmed using Kraken2 (Standard database enriched with protozoa, fungi, and plant genomes) (41), Diamond (nr database, March 2023), PLAST (nt database, June 2023), and BLAST (nt database, March 2023) and MyCC binning results. The ability to recover these identified contigs by the different methods was examined.

### Performance measures for eukaryotic genome recovery

We evaluated Eukfinder (short and long) and compared it with Refmapping, EukRep, and Tiara for recovering *Blastocystis* genomes from mock communities and human gut metagenome samples. Genome completeness was assessed using QUAST v5.0.2 by total genome length and fraction of the *Blastocystis* genome recovered. A set of SCGs was identified in the ST1 cleaned genome and used for the SCG test (1,943 genes, File S4). The completeness of human gut metagenome samples was also assessed using BUSCO 4.0.6 with the stramenophiles_odb10 and eukaryote_odb10 data sets (25, 26). Performance measures were compared using pairwise Student’s *t*-tests (α < 0.05), with Bonferroni correction. Eukfinder_short was compared with Refmapping, and Eukfinder_long was compared with EukRep and Tiara.

### Mitochondrial genome recovery

We assessed the recovery of mitochondrial genomes from the identified binned reads of each method. Eukfinder and EukRep classify *Blastocystis* mitochondrial contigs as eukaryotic, whereas Tiara directly identifies mitochondrial contigs. Mitochondrial reference genomes for ST3 (NC_018042.1) and ST4 (NC_027962.1) were used for Refmapping to map and assemble mitochondrial reads.

## Data Availability

Eukfinder is developed and implemented in Python. The Eukfinder source code is available in the GitHub repository at https://github.com/RogerLab/Eukfinder. Default reference databases can be downloaded from https://perun.biochem.dal.ca/Eukfinder. The binning process is available at https://github.com/RogerLab/MetagenomeBinningWorkflow.

## SUPPLEMENTAL MATERIAL

File S1 mbio.00699-25-s0001.xlsx Quast analysis of mock community tests.

File S2 mbio.00699-25-s0002.xlsx Contamination contig precision and recall analyses.

File S3 mbio.00699-25-s0003.xlsx Quast and Busco results from the human gut samples.

File S4 mbio.00699-25-s0004.xlsx Accession numbers of 1,943 single-copy genes in *Blastocystis* ST1 cleaned genome.

File S5 mbio.00699-25-s0005.xlsx Genomes used in custom Centrifuge database.

File S6 mbio.00699-25-s0006.xlsx Genomes used in custom PLAST database.

Supplemental Material mbio.00699-25-s0007.pdf Supplemental methods, figures, tables, file captions, and references.

ASM does not own the copyrights to Supplemental Material that may be linked to, or accessed through, an article. The authors have granted ASM a non-exclusive, world-wide license to publish the Supplemental Material files. Please contact the corresponding author directly for reuse.
