# Supplementary material for: Eukfinder: a pipeline to retrieve microbial eukaryote genome sequences from metagenomic data
Source: mBio. 2025 Apr 10;16(5):e00699-25. doi: 10.1128/mbio.00699-25 (PMC12077102; doi:10.1128/mbio.00699-25)
Supplement: Supplemental Material — Supplemental methods, figures, tables, file captions, and references. [file mbio.00699-25-s0007.pdf]

Eukfinder: a pipeline to retrieve microbial eukaryote genome sequences from metagenomic data

Dandan Zhao[¥,a,b], Dayana E. Salas-Leiva[¥,a,c], Shelby K. Williams[a,b], Katherine A. Dunn[a,b], Jason Shao[a,b], and Andrew J. Roger[a,b,*]

[a] Institute for Comparative Genomics, Dalhousie University, Nova Scotia, Canada

[b] Department of Biochemistry and Molecular Biology, Dalhousie University

[c] Department of Biochemistry, Cambridge University, CB2 1QW, UK

[¥] These authors contributed equally to this work.

*Corresponding author: andrew.roger@dal.ca

SUPPLEMENTARY METHODS

Metagenomics enables the exploration of the functions, physiologies, and evolutionary histories of both prokaryotic and eukaryotic microbes in various ecosystems through whole-genome shotgun (WGS) sequencing. However, despite their ecological significance, studies on microbial eukaryotes using metagenomics have lagged behind those on prokaryotes due to the challenges in identifying and assembling high-quality eukaryotic genomes from WGS data. To tackle this issue, we have created Eukfinder, a bioinformatics pipeline designed to recover and assemble nuclear and mitochondrial genomes of eukaryotic microbes from WGS metagenomic data. Eukfinder employs two specialized, customizable databases to classify reads taxonomically, tailoring the process to the specific dataset or environment. We applied Eukfinder to human gut microbiome WGS metagenomic data to recover genomes of the protistan parasite *Blastocystis* sp., a common inhabitant of the gastrointestinal tracts of humans and animals. We validated Eukfinder with both simulated gut microbiome datasets, which varied in the number of Blastocystis reads mixed with bacterial reads, and real metagenomic gut samples containing Blastocystis. The performance of Eukfinder was compared with other established workflows. Given sufficient reads, Eukfinder effectively assembles high-quality, near-complete nuclear and mitochondrial genomes from diverse *Blastocystis* subtypes without needing a reference genome. Moreover, with adequate sequencing depth, Eukfinder surpasses similar tools in recovering eukaryotic genomes from metagenomic data. Thus, Eukfinder is a valuable tool for the reference-independent, cultivation-free study of eukaryotic microbial genomes from environmental metagenomic sequencing samples.

Database preparation

Two specialized databases used in the Eukfinder classification pipeline include genomes from bacteria, archaea, eukaryotes, viruses, mitochondria, and EukPathDB, providing

comprehensive taxonomic coverage. The number of genomes in each category is listed in Table S11.

Specialized Centrifuge database (DB1)

Centrifuge (1) is a metagenomics taxonomy classification software tool that uses an optimized indexing scheme which allows for the rapid classification of sequencing reads. It contains built-in tools to download genomes from the National Center for Biotechnology Information (NCBI) website and to build custom databases. To maximize Centrifuge's ability to classify gut metagenome data and eukaryote genomes in particular, a custom database was built (here referred to as specialized DB1). Archaeal, bacterial, and viral genomes associated with the gut microbiome, or without any specific environment listed in the project names, were downloaded from NCBI (as of Jan 2019). Genomes from all four assembly levels (complete, chromosome, scaffolds, and contigs) were included. An in-house python script was applied to exclude genomes retrieved from environments other than the GI tract (marine, soil, or freshwater). In addition, 4,930 species-level bacterial and archaeal genome bins from >9000 human metagenomes (2) and 913 microbial genomes obtained from rumen metagenomic sequencing (3) were downloaded. Redundant bacterial and archaeal genomes were removed with GTDB-Tk (4) and Treemmer (5). Viral genomes were clustered using MyCC (6) and 40% of the contigs from each cluster (minimum 20 contigs per cluster) were randomly chosen to be included in the database. EupathDB (7) were also included and any pre-downloaded NCBI genomes for those same species were excluded. Additional eukaryotic genomes for protists, fungi, and animals with complete or chromosome level genome assemblies and their corresponding mitochondrial genomes were downloaded from NCBI Genbank (for full list of genomes used see Supplementary File 5).

In addition, all available genome sequences of *Blastocystis* (Table S1) were also included in DB1, after a decontamination step consisting of mapping the *Blastocystis* genomes against the NCBI nucleotide (nt) database (as of Jan 2019), which did not include any known *Blastocystis* sequences. Contigs in the *Blastocystis* reference genomes that matched >50% of their total length to a bacterium, archaeon, or viral sequence in the nt database and had a nt identity ≥ 80% were considered contaminants and eliminated from the draft genomes used to create specialized DB1. For a full list of excluded *Blastocystis* contigs see Table S12. In-house python scripts were used to build the index files and the centrifuge-build command from Centrifuge was used to construct specialized DB1.

Specialized PLAST database (DB2)

PLAST (Parallel Local Alignment Search Tool) (8) is a rapid sequence similarity search tool that is more sensitive though not as fast as Centrifuge. To mitigate computational burden, a specialized PLAST database (hereafter referred to as specialized DB2) was built with a subset of reference genomes from archaea, bacteria, eukaryotic, and mitochondrial genomes selected from the complete set of all the downloaded genomes (Supplementary File 6). Specialized DB2 overlaps with specialized DB1 to some degree to enhance the sensitivity of the classification

method since PLAST search results were based on similarity (identity) while centrifuge only reports exact alignments with the minimal hit length. For viruses, all viral genomes were downloaded from NCBI Refseq database (ftp.ncbi.nlm.nih.gov/refseq/release/viral/, Mar 2019). All the genome files were combined into a single fasta file, and the database was built using BLAST (9) "makeblastdb" command and a simplified index file containing information that cross-references each sequences accession entry in the database to its respective taxonomic group (i.e., bacteria, archaea, eukaryote, and virus).

Database with limited or no Blastocystis

Comparative genome analysis had revealed that there is a great diversity among the genomes of ST1, ST4 and ST7 and the percentage of unique protein-coding genes ranges from 6% to 20% (10), which likely is also the case for other *Blastocystis* genomes in GenBank. Therefore, a combination of all available *Blastocystis* genomes except ST1 (including ST2-ST4, ST6-ST9 genomes, noted as *Blastocystis* ST2-9) was used as reference genomes for the Refmapping method. For Eukfinder, we created two custom Centrifuge and PLAST databases that were either missing the *Blastocystis* ST1 genome (which includes *Blastocystis* ST2-9 genomes) or were missing all *Blastocystis* genomic information. These two databases are referred to as woST1 and woBlasto, respectively.

To examine the performance of Eukfinder_short in recovering genomes when no reference was available, we created two modified versions of both DB1 and DB2. In one modification we created both databases without *Blastocystis* ST1 genome (hereafter referred to as woST1) and in the second modification we removed all *Blastocystis* genomes from DB1 and DB2 (hereafter referred to as woBlasto). We then reran the mock datasets using these databases with Centrifuge and PLAST. In addition, we reran Refmapping replacing the ST1 reference genome with published *Blastocystis* sp. ST2-4, 6-9 as the reference as a parallel test to simulate the situation when there is no direct reference genome.

Databases Used for Binning and Contig Validation

Several external databases were utilized in the binning workflow to validate contigs and enhance binning accuracy. These databases supported the identification and classification of sequences into taxonomic groups and ensured the accuracy of the final genome assemblies.

The Metaxa2 LSU/SSU databases (11), based on SILVA release 111, were employed to identify ribosomal RNA (rRNA) sequences, including large subunit (LSU) and small subunit (SSU) rRNA genes. These databases were vital for distinguishing eukaryotic, bacterial, and archaeal sequences, aiding in the accurate classification of contigs during the binning process.

The PLAST nt database (January 2021 release) was downloaded from NCBI (https://ftp.ncbi.nlm.nih.gov/genomes/refseq/). Similarly, the BLAST database (March 2023 release) was downloaded from NCBI and built using the default makeblastdb command.

Additionally, we utilized the Kraken2 (12) database PlusPFP, an enriched version of the standard Kraken2 database. The PlusPFP database combines the standard database (including RefSeq archaea, bacteria, viral, plasmid, human1, and UniVec_Core sequences) with genomes from protozoa, fungi, and plants to improve sensitivity for eukaryotic content. The database was downloaded from the Kraken2 AWS-indexes repository at https://benlangmead.github.io/aws-indexes/k2.

Identifying optimal parameters for alternative methods

To ensure optimal results from all methods, we first identified the parameters for each program that maximized genome completeness and contiguity. We investigated three of the *Blastocystis* read count mock communities (500K, 1M, 2M) to find the best parameters for three methods that would be used to analyze all datasets.

EukRep is a bioinformatics tool designed to identify and classify eukaryotic sequences from complex metagenomic datasets by leveraging k-mer compositions and linear Support Vector Machine (SVM) algorithms, enabling the recovery and assembly of high-quality eukaryotic genomes (13). It offers three stringency modes: strict, balanced, and lenient. Additionally, the '-tie' flag decides how to classify sequences with equal eukaryotic and prokaryotic predictions. We compared genomes recovered with all three stringency modes and one lenient mode with the '-tie' set to prokaryotic. The results showed that the lenient stringency provided the most complete genome recovery at all read counts tested (Fig. S14). The strict mode, which was designed to have a lower false positive rate generated the least complete genomes and lenient modes the most complete genomes with balanced mode intermediate. On average, the balance mode increased the completeness by 0.5-1.5% more than the strict mode; the lenient mode further increased the completion 2-5%, while setting "tie" decision handling to prokaryotic, generated genomes 1% less complete than when the default (eukaryotic) was used. Based on these findings, we ran EukRep with lenient mode and default eukaryotic "tie" decision for all datasets for comparison with Eukfinder_long.

Tiara is a deep-learning-based classification system designed to identify eukaryotic sequences in metagenomic datasets, performing a two-step classification to separate nuclear and organellar eukaryotic fractions and further dividing organellar sequences into plastidial and mitochondrial genomes (14). Tiara uses neural networks to sort metagenomic reads into taxonomic categories. Its workflow parameters include k-mer size (DNA substring size) and probability threshold (minimum score for considering results). To examine these parameters Tiara was tested under a combination of three different k-mers (4-mer, 5-mer, and 6-mer) and five different probability thresholds (p=0.5, 0.55, 0.6, 0.65, 0.7). We found that utilizing a k-mer size of 4 and probability threshold of 0.7 produced the most complete and contiguous genomes (Fig. S15). Therefore, Tiara was run with a k-mer of 4 and a probability threshold of 0.7 for all datasets for comparison with Eukfinder_long.

Beghini and colleagues applied a bioinformatic method of reference-genome-based read mapping and assembly to construct *Blastocystis* genomes from gut metagenomic datasets

(hereafter referred to as ‘Refmapping’) (15). Refmapping maps metagenomic reads to the reference genome of interest. Refmapping with Bowtie2 has two different strategies: global (end-to-end) and local mapping. End-to-end mapping requires complete read alignment to the reference, while local alignments allow partial read matches to optimize the alignment score. We examined the performance of both under three test groups of mock community datasets (500K, 1M, 2M of random selected *Blastocystis* reads) and found that local alignments generated more complete and contiguous genomes compared to global (end-to-end) alignments (Fig. S16). This was especially true when there were lower numbers of reads to map to the reference genome. With 500 K reads, the genomes recovered using the local alignment had an average completion of >60% while the genomes recovered using global alignment were <20% complete. When the number of *Blastocystis* reads increased to 1M, the genomes recovered using the local alignment had an average completion of 90% while the genomes recovered using global alignment were <60% complete. Therefore, we ran Refmapping with local alignment on all datasets and used the *Blastocystis* ST1 genome as the reference sequence for the mock community and ST3 and ST4 genomes for the human metagenome data, for comparison with Eukfinder_short.

Analysis of metagenome sample from the Mediterranean Sea (Tara Oceans)

Metagenomic data from a Mediterranean Sea site (SRA: ERR868402) of the Tara Oceans Initiative (16), was analyzed to assess Eukfinder’s classification performance. This dataset originated from the 0.8–20 µm size fraction and contained 190,669,700 reads. Most reads (up to 93%) were inferred to belong to Bacteria, Archaea, or Virus. Reads were assembled using metaSPAdes-3.15.5 with default parameters (16) which resulted in 64,535 contigs ≥1000 bp. As the true classification of these contigs is unknown, multiple independent classifiers were used in order to infer the accuracy of eukaryotic classifications by Eukfinder_long, EukRep, and Tiara. This involved examining contigs ≥1000 bp with Kraken2 (PlusPFP: Standard plus Refeq protozoa, fungi & plant), Diamond (17) (nr database, March 2023), PLAST (nt database, Jan 2021), and BLAST (nt database, March 2023), and a taxonomic category was assigned to contig if at least two tools provided the same classification. Classification could be inferred this way in 53,411 contigs, with 3,733 of those inferred to be Eukaryota. The remaining 11,124 were unclassified or conflicted results by different methods. Using only those contigs that had inferred classification we evaluated the three methods (Eukfinder_long, EukRep, and Tiara) performances in accurately identifying eukaryotic content. Contigs were classified as True Positives, False Positives, or False Negatives based on agreement with inferred classification as outlined above to evaluate the performance of the three methods. Eukfinder_long was tested with two sets of parameters: strict parameters for PLAST (e-value = 0.01, 70% identity, 20% hit length coverage) and Centrifuge (minimum hit length 40bp) and lenient parameters for PLAST (e-value = 0.01, 50% identity, 10% hit length coverage) and Centrifuge (minimum hit length 25bp) (TABLE S9). EukRep was tested using default, balance mode; Tiara with --k1 4 --k2 6, -p 0.7 0.65.

Additional Supplementary Files

Supplementary File 1. Quast analysis of Mock community tests.xlsx

Supplementary File 2. Contamination contigs Precision and recall analyses.xlsx

Supplementary File 3. Quast and Busco results from the human gut samples.xlsx

Supplementary File 4. Accession Numbers of 1943 Single-Copy Genes in Blastocystis ST1 Cleaned Genome.xlsx

Supplementary File 5. List of genomes used in custom Centrifuge database.xlsx

Supplementary File 6. List of genomes used in custom PLAST database.xlsx

SUPPLEMENTARY FIGURES

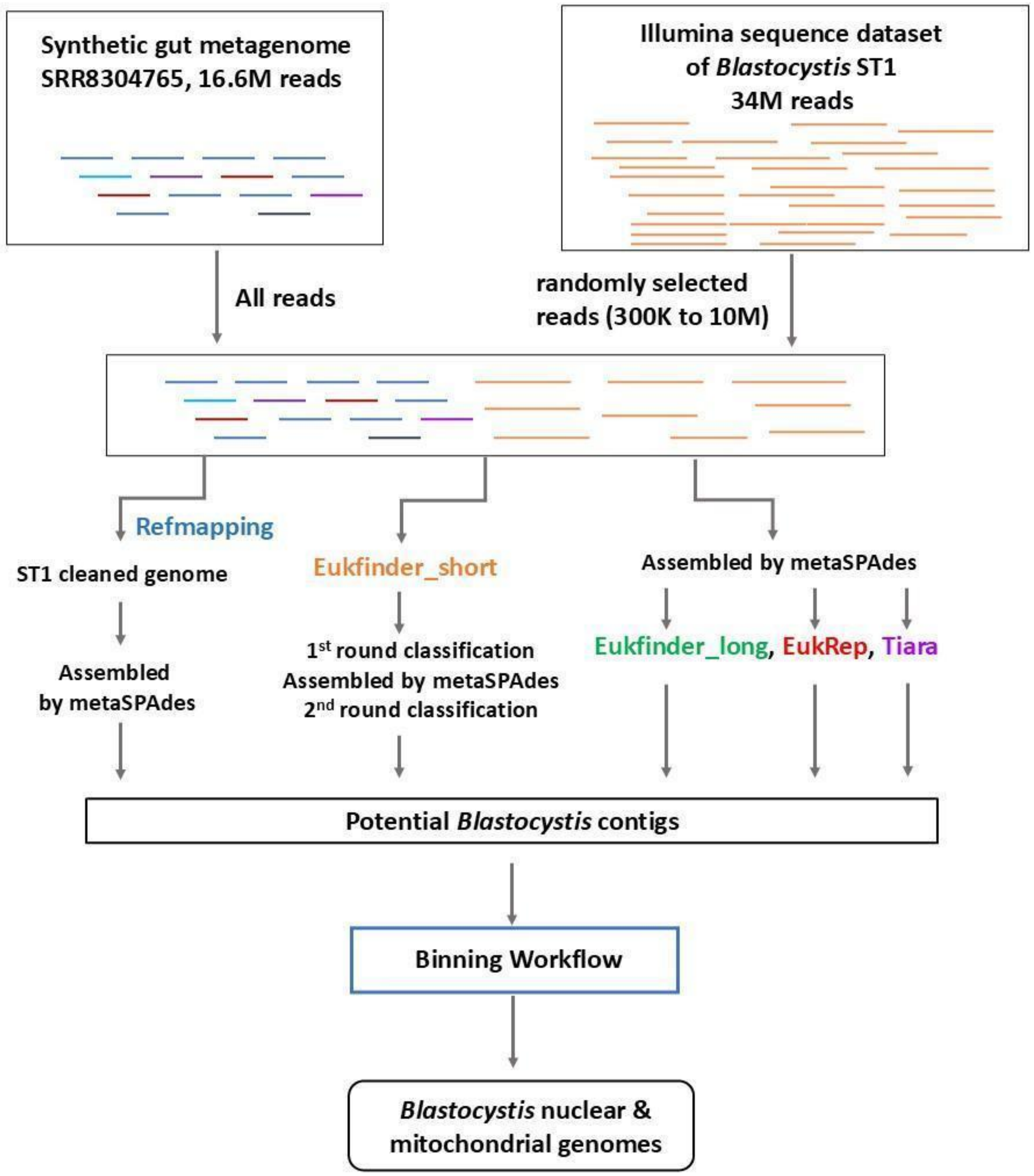

Figure S1. Schematic representation of mock community dataset preparations and analyses. The reads from synthetic gut metagenome SRR8304765 were combined with randomly selected *Blastocystis* ST1 reads (300K – 10M) to create the mock community datasets analyzed by the methods. The workflow outline for each method is outlined. Note the same binning method was used on the recovered contigs from each method.

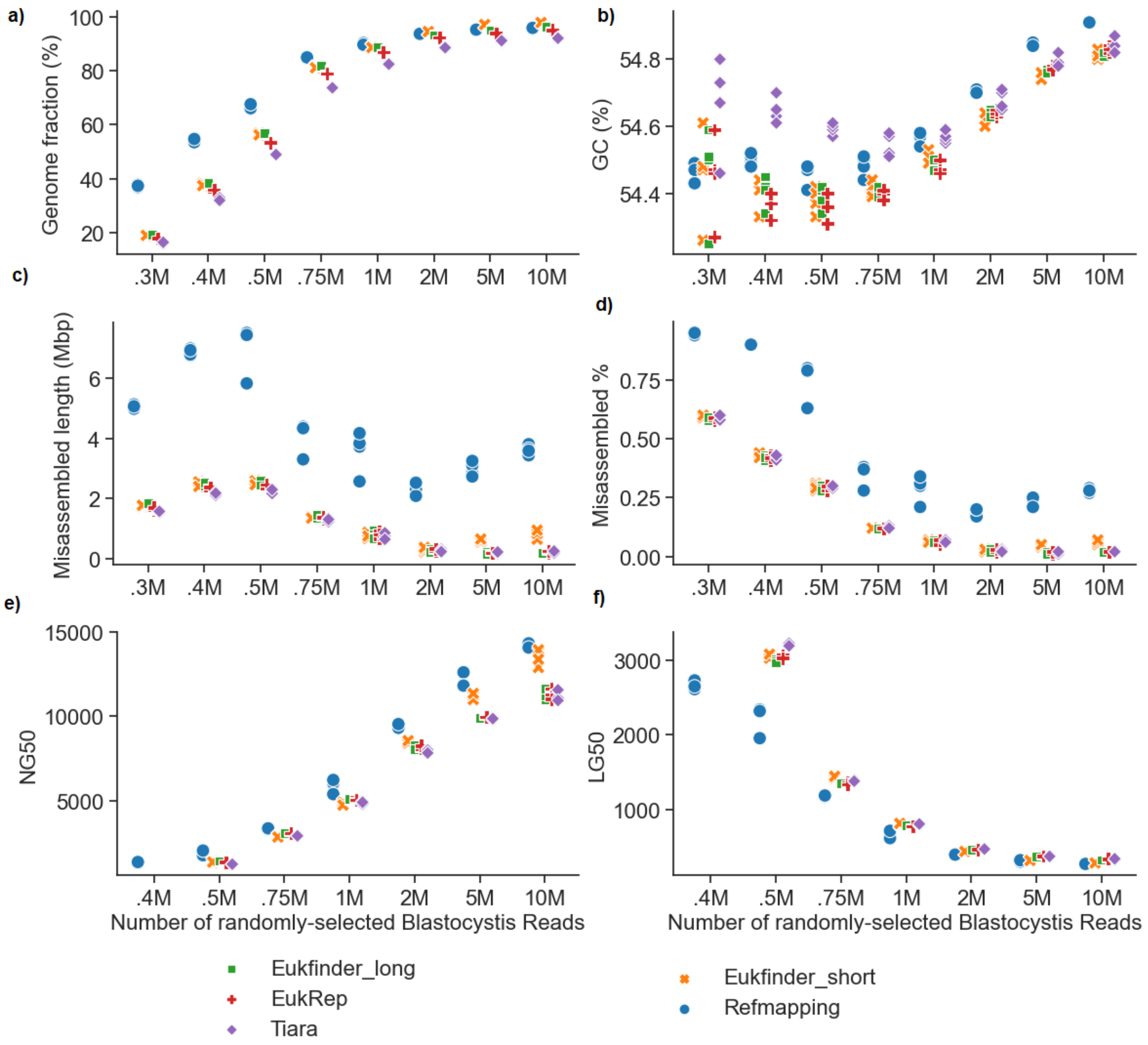

Figure S2. Comparison of methods (Eukfinder_short, Refmapping, Eukfinder_long, EukRep, and Tiara) in recovering *Blastocystis* ST1 genomes in eight mock metagenomic datasets (staggered mix-51 and varying amounts of randomly selected *Blastocystis* ST1 Illumina sequencing reads). Plot of QUAST assessment of methods recovery of *Blastocystis* ST1 for the mock community datasets, a) percent of reference genome recovered, b) percent GC, c) misassembled contig length (Mbp), d) percentage of misassembled length relative to the total length of recovered genomes, e) NG50 and f) LG50. Note NG50 and LG50 were not computable for all of 300K and some of 400K datasets. EukRep was run in lenient mode, Tiara was run with kmer of 4 and probability threshold of 0.7. Refmapping was run in local mode using *Blastocystis* ST1 genome as the reference genome. Detailed QUAST results are shown in Supplementary File 1.

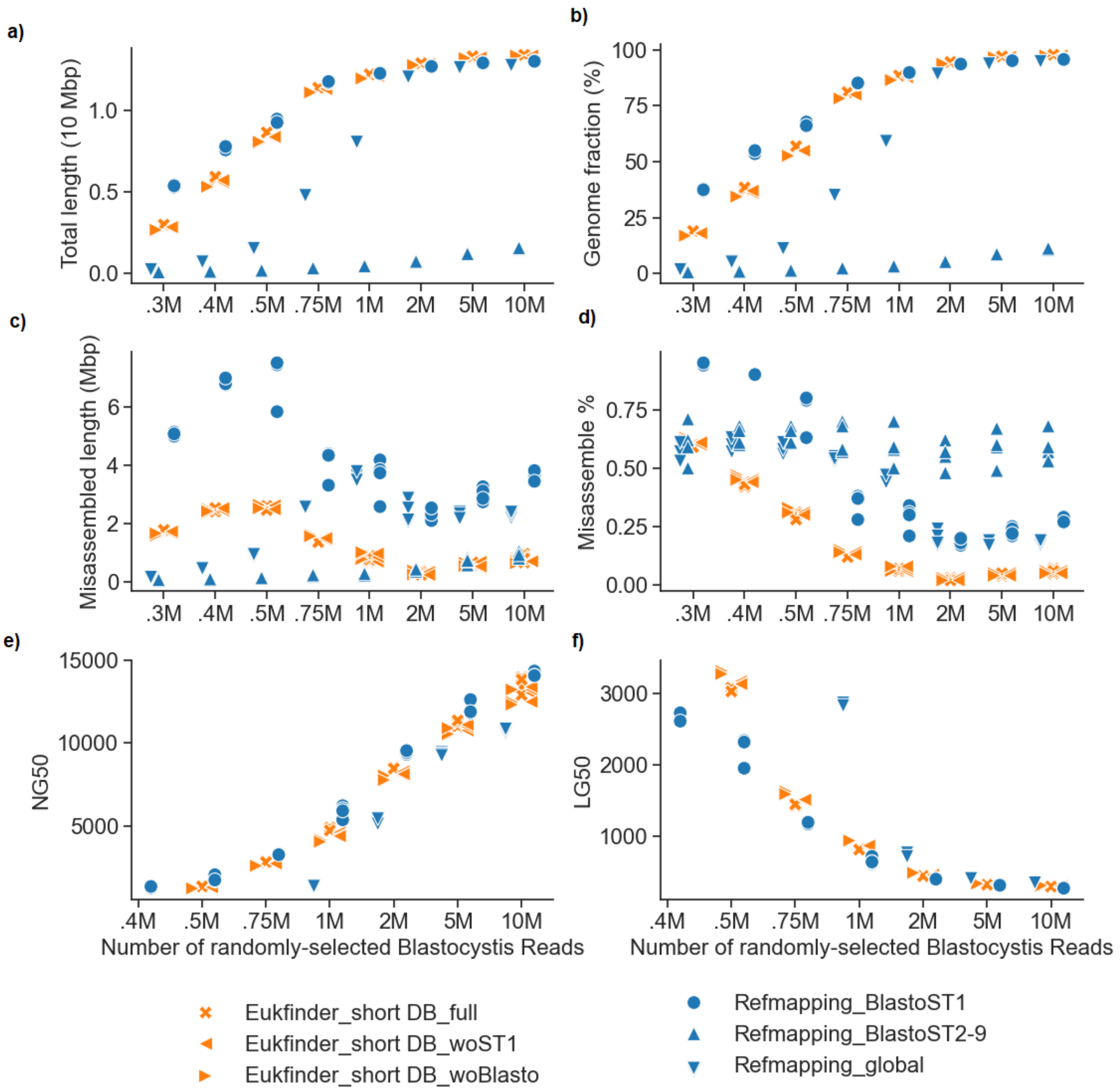

Figure S3. Eukfinder_short and Refmapping performance with and without inclusion of the ST1 genome, assessed by QUAST compared to ST1 reference genome, a) Total length (Mbp), b) genome fraction recovered (%), c) misassembled contig length (Mbp), d) percentage of misassembled length relative to the total length of recovered genomes, e) NG50 and f) LG50. Note NG50 and LG50 were not computable for all of 300K and some of 400K datasets. Eukfinder_short was assessed under three sets of databases: the complete database (noted as full), the database without *Blastocystis* ST1 genome (using *Blastocystis* ST2-ST4, ST6-ST9 genomes noted as woST1) and no *Blastocystis* genomes (noted as woBlasto). Refmapping was assessed under two scenarios, including ST1 as the reference (noted as BlastoST1) or using all available *Blastocystis* genomes except ST1 (noted as BlastoST2-9) as the reference. The impact of using global alignment on Refmapping’s ability to recover the genome is also plotted.

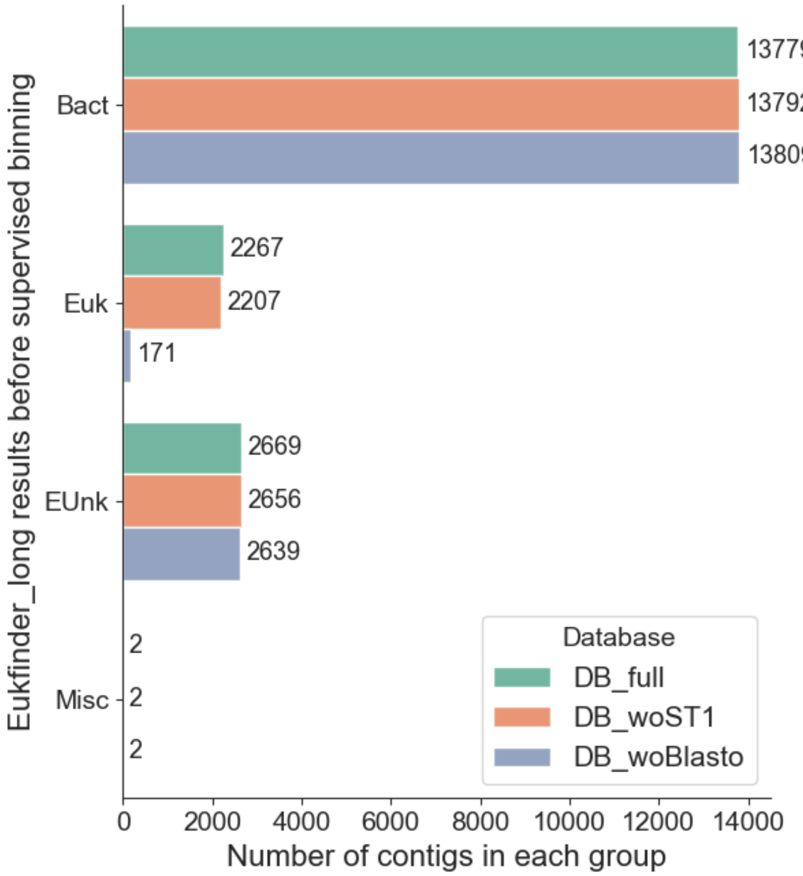

Figure S4. Classification results for a mock community sample containing 2 million *Blastocystis* reads by Eukfinder_long using different database configurations; DB_full (complete database), DB_woST1 (database excluding *Blastocystis* ST1 genome), and DB_woBlasto (database excluding all *Blastocystis* genomes). Bar plot shows the distribution of contigs into taxonomic categories; bacteria (Bact), eukaryotic (Euk), eukaryotic and unknown combined (EUnk), and miscellaneous (Misc, including viruses), prior to binning. The number of contigs in each category is indicated.

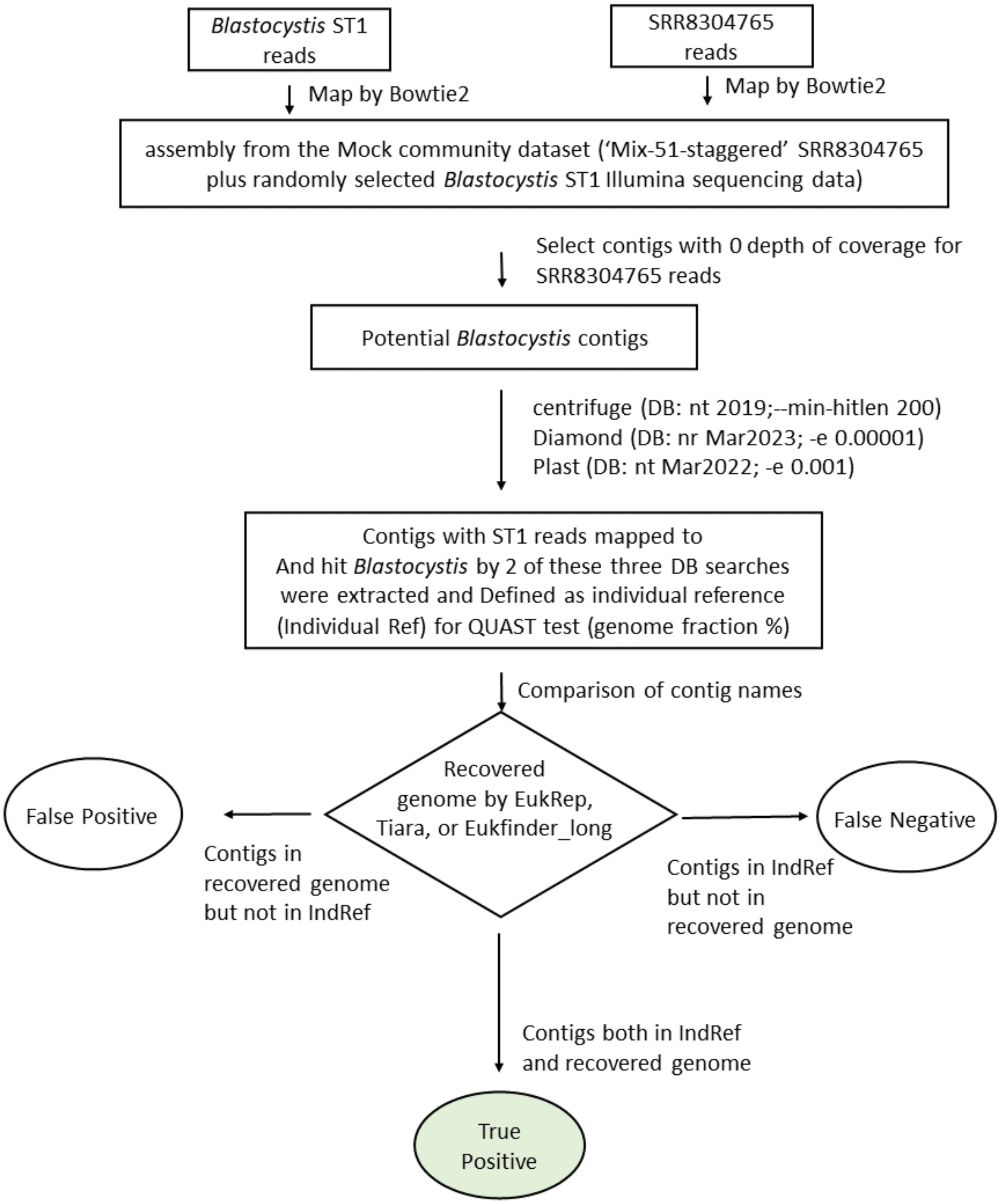

Figure S5. Schematic representation of precision and recall analyses. Analyses were performed on the assembled contigs of each read dataset which were compared with the original 34M *Blastocystis* ST1 reads and bacterial reads of SRR8304765. Contigs without any SRR8304765 reads mapped were considered as potential Blastocystis contigs. Contigs with *Blastocystis* ST1 reads mapped and identified as *Blastocystis* by two of the three tools (Centrifuge, Diamond and PLAST) were noted as true *Blastocystis* contigs. These contigs were referred to as Individual reference *Blastocystis* genome for each tested dataset and used as the reference genome (Individual Ref) in QUAST for Figure 3.

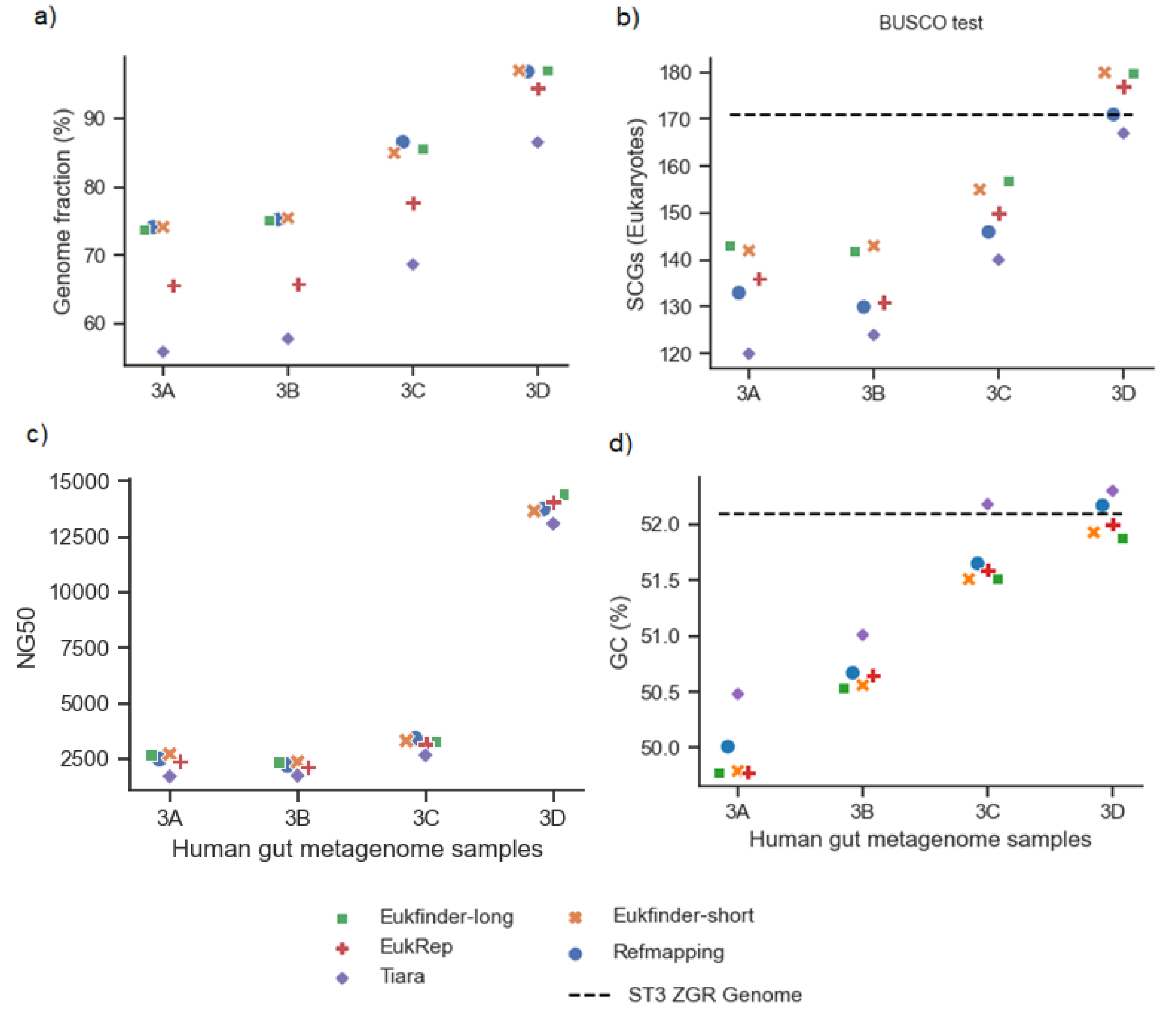

Figure S6. *Blastocystis* ST3 genomes recovered from four human gut metagenome samples (3A-D) using Refmapping, Eukfinder_short, Eukfinder_long, EukRep and Tiara. Completeness of ST3 genomes in samples based on QUAST analyses, a) genome fraction recovered (%); b) total number of SCGs detected (complete and fragmented) using eukaryote_odb10; c) NG50, d) percent GC content. EukRep was run using lenient setting, Tiara was run with kmer of 4 and probability threshold of 0.7 and Refmapping was run in local mode using *Blastocystis* ST3 ZGR as the reference genome. Dashed line represents *Blastocystis* ST3 ZGR reference genome number for comparison. Detailed QUAST results are shown in Supplementary File 3.

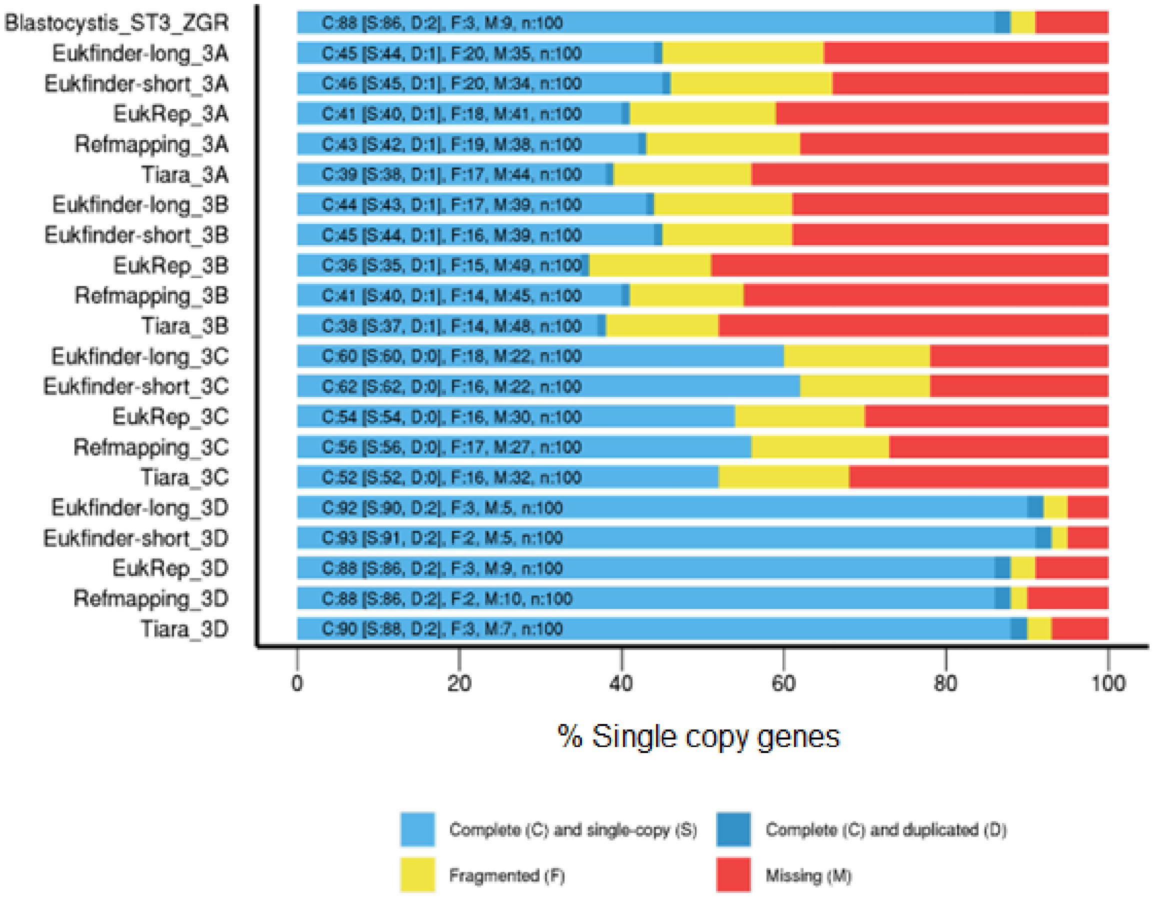

Figure S7. BUSCO assessment of *Blastocystis* ST3 genomes recovered from four human gut metagenome samples (3A-3D) using Refmapping, Eukfinder_short, Eukfinder_long, EukRep and Tiara using stramenopiles_odb10.

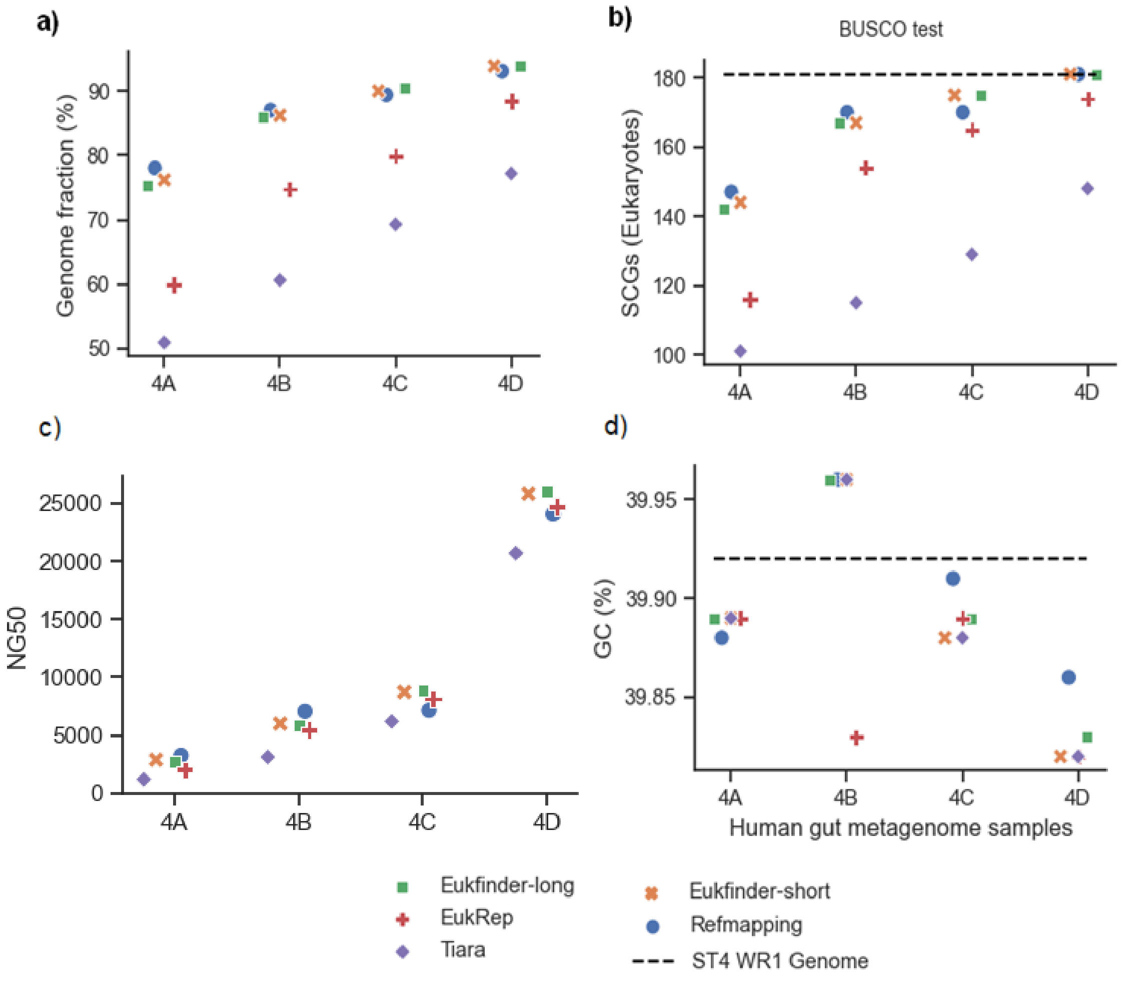

Figure S8. *Blastocystis* ST4 genomes recovered from four human gut metagenome samples (4A-D) using Refmapping, Eukfinder_short, Eukfinder_long, EukRep and Tiara. Completeness of ST4 genomes in samples based on QUAST and BUSCO analyses; a) genome fraction recovered (%); b) number of SCGs (complete and fragmented) using eukaryote_odb10; c) NG50, d) percent GC content; and e) bar plot of SCGs using stramenopiles_odb10. EukRep was run using lenient setting, Tiara was run with kmer of 4 and probability threshold of 0.7 and Refmapping was run in local mode using *Blastocystis* ST4 WR1 as the reference. Reference genome numbers for *Blastocystis* ST4 WR1 (dashed line) are shown for comparison. Detailed QUAST results are shown in Supplementary File 3.

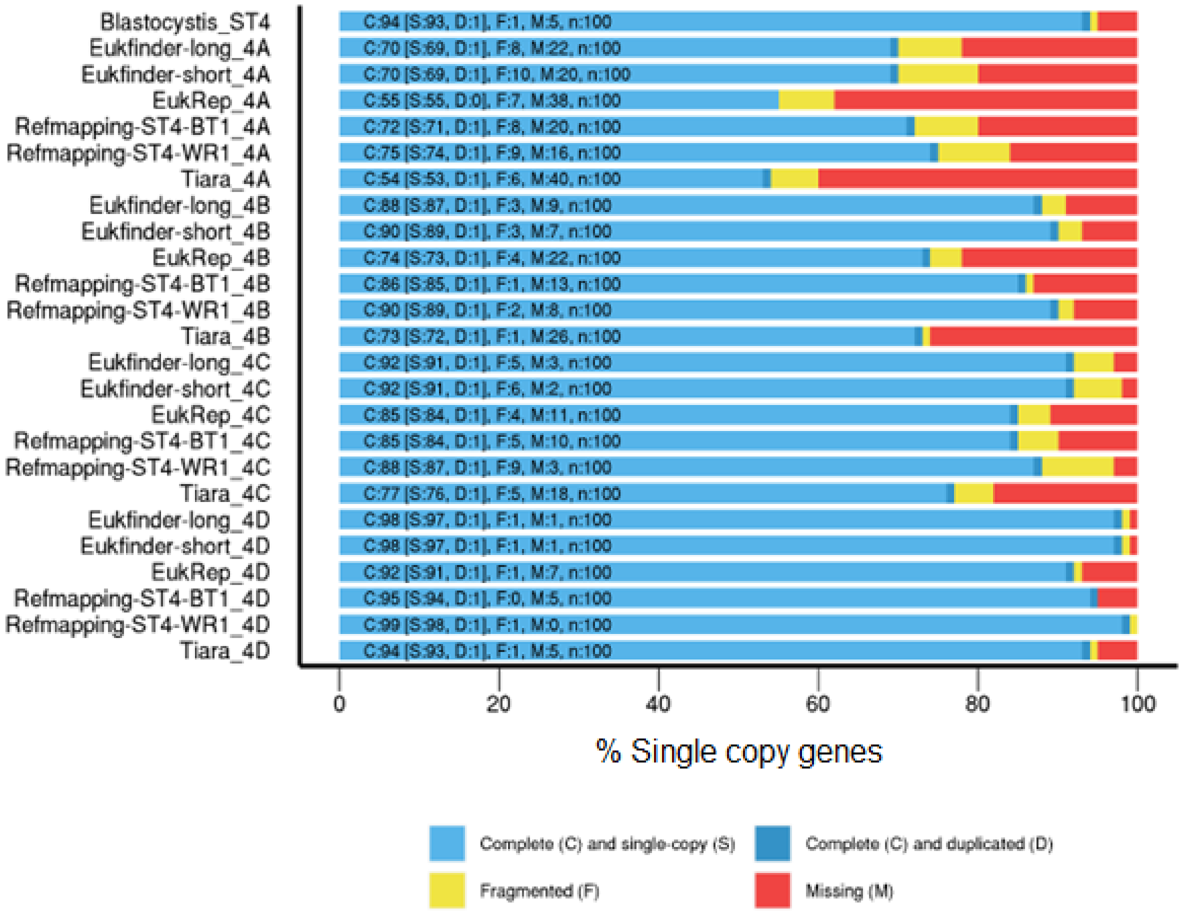

Figure S9. BUSCO assessment of *Blastocystis* ST4 genomes recovered from four human gut metagenome samples (4A-4D) using Refmapping, Eukfinder_short, Eukfinder_long, EukRep and Tiara using stramenopiles_odb10.

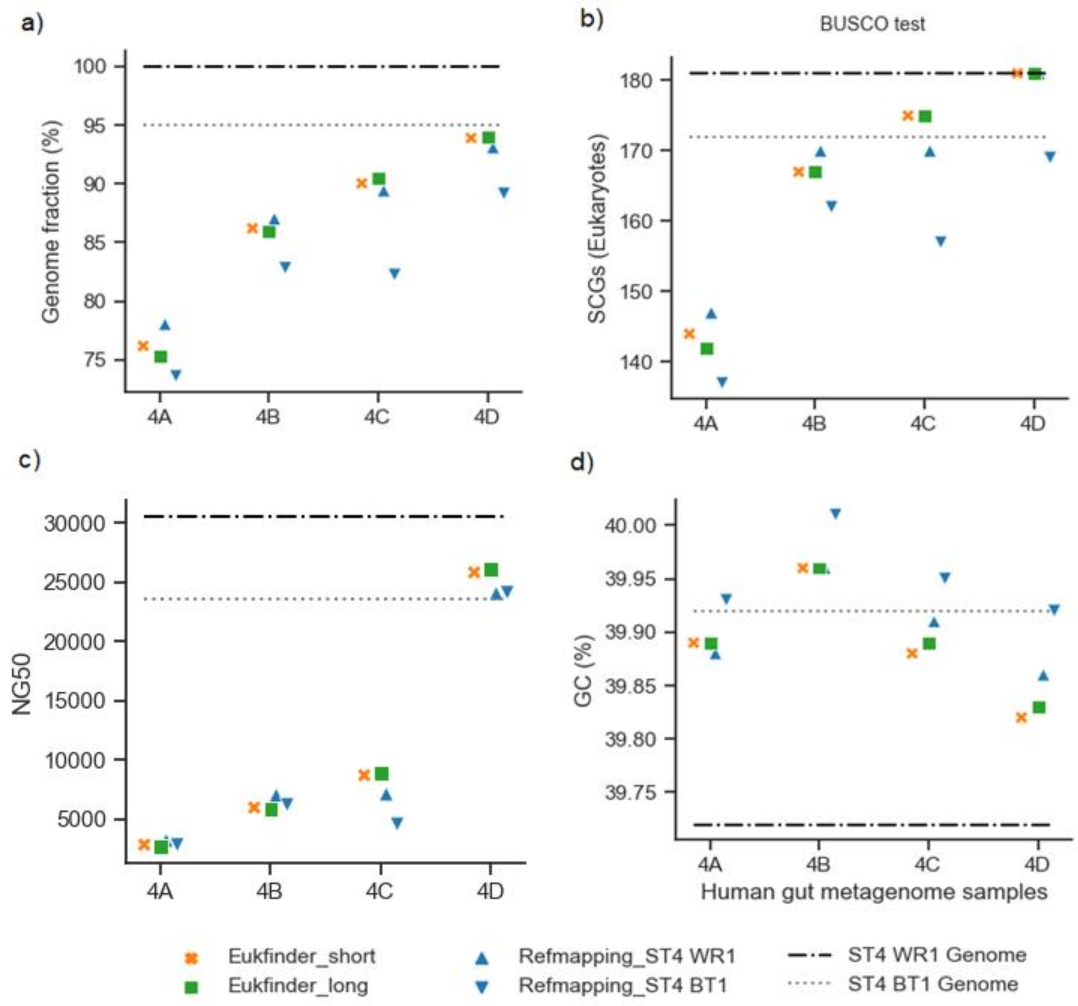

Figure S10. Examining the impact of different ST4 reference genomes on *Blastocystis* ST4 genomes recovered from four human gut metagenome samples (4A-D) using Refmapping, with comparison to Eukfinder_short, and Eukfinder_long. Completeness of ST4 genomes in samples based on QUAST and BUSCO analyses; a) genome fraction recovered (%); b) number of SCGs (complete and fragmented) using eukaryote_odb10; c) NG50; and d) percent GC content. Refmapping was run in local mode using *Blastocystis* ST4 WR1 (triangle) or BT1 (upside down triangle) as the reference genomes. Reference genome numbers are shown for comparison for *Blastocystis* ST4 WR1 (dashed-dot line) and *Blastocystis* BT1 (dotted line). Detailed QUAST results are shown in Supplementary File 3.

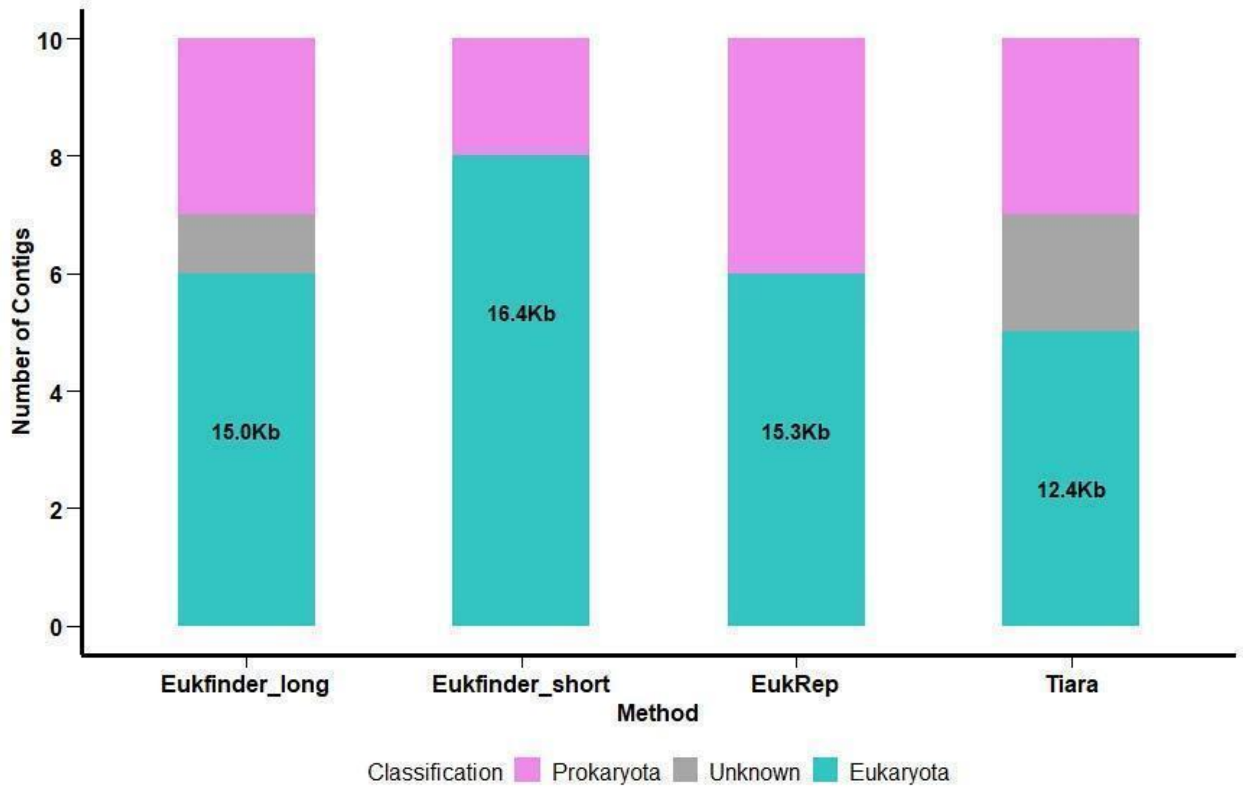

Figure S11. Classification of 10 contigs inferred to be *Saccharomyces cerevisiae* from human gut metagenome sample 4C using Eukfinder_long, Eukfinder_short, EukRep, and Tiara.
Total lengths of contigs classified as Eukaryota are indicated within the teal bars.

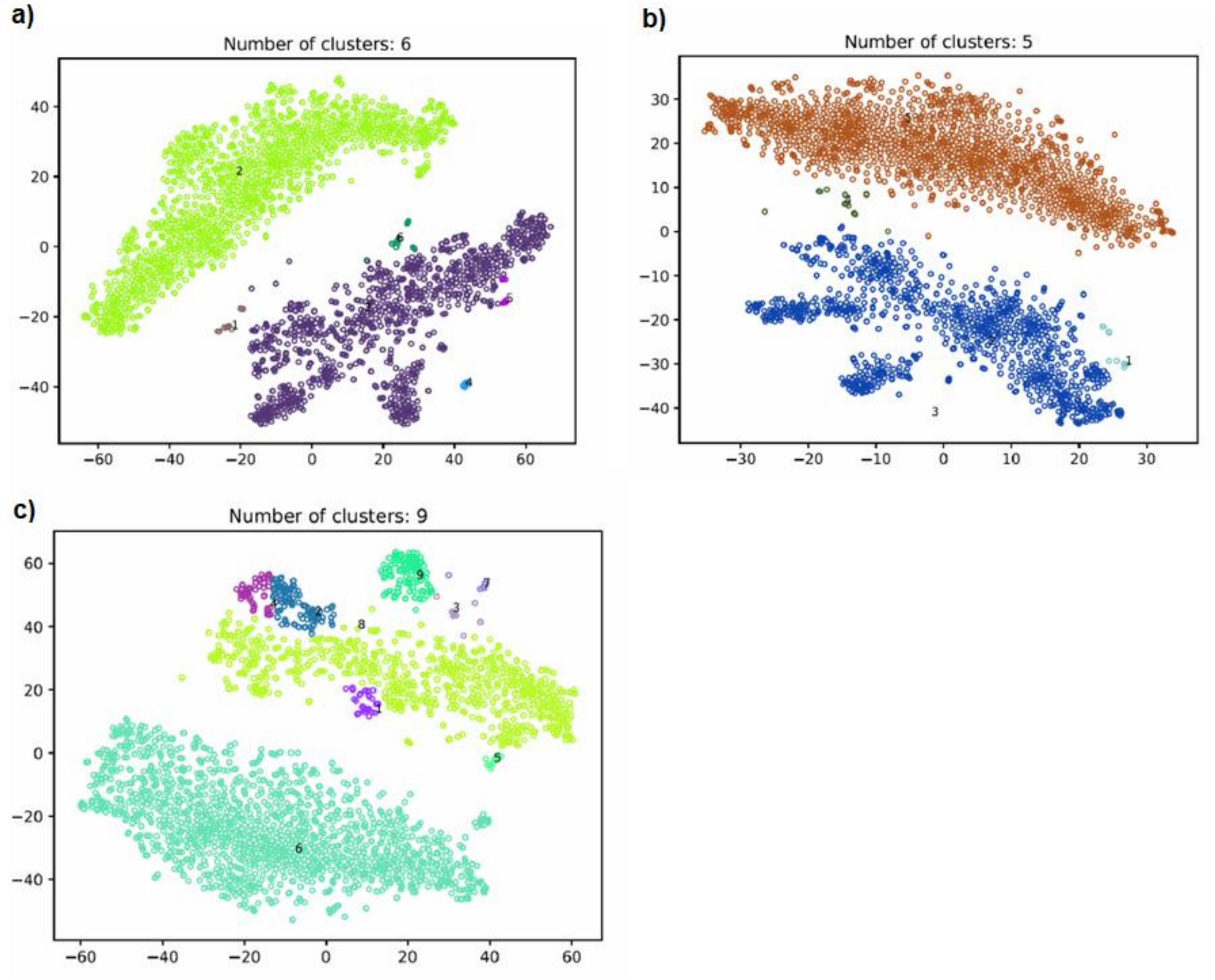

Figure S12. Example of Genome cluster maps generated by MyCC using three k-mer settings: a) 4-mer, b) 5-mer, and c) 5mer and 6-mer. Each circle represents a contig. Each cluster is shown in a different color and represents a possible genome. Note: the colors between images are independent.

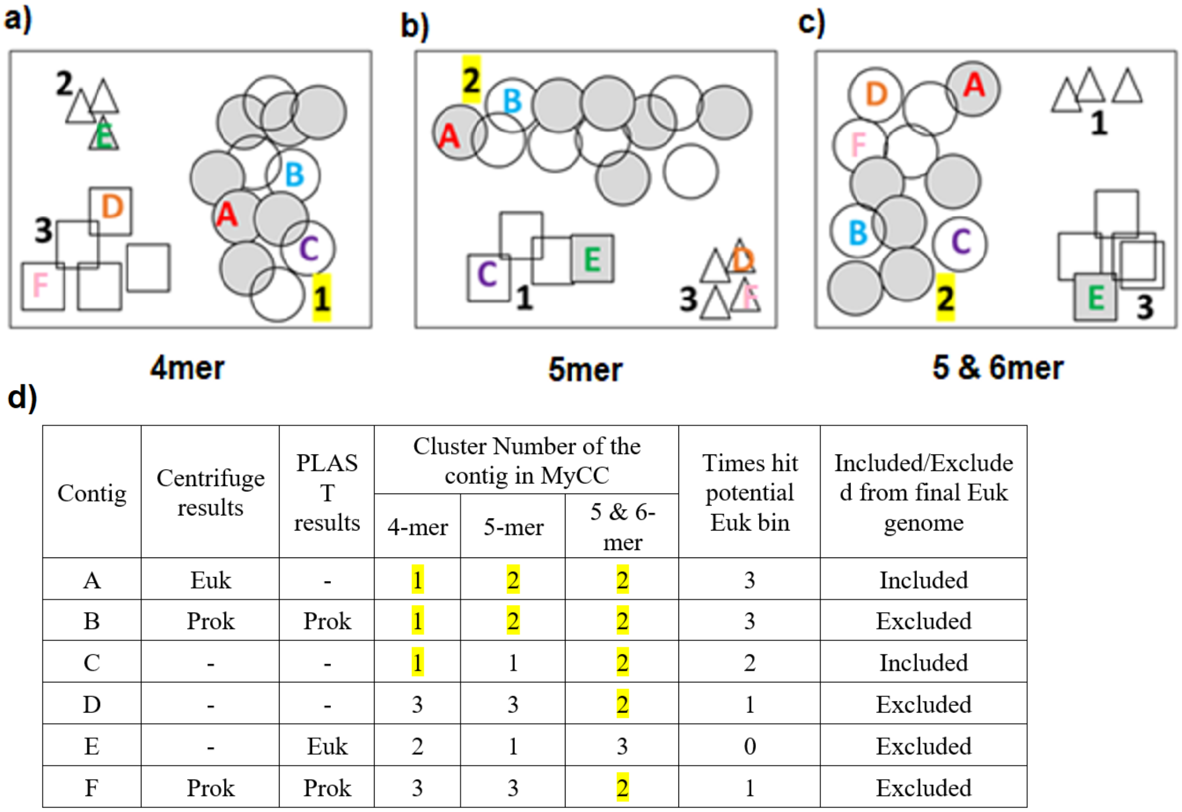

d)

Contig	Centrifuge results	PLAST results	Cluster Number of the contig in MyCC			Times hit potential Euk bin	Included/Excluded from final Euk genome
			4-mer	5-mer	5 & 6-mer		
A	Euk	-	1	2	2	3	Included
B	Prok	Prok	1	2	2	3	Excluded
C	-	-	1	1	2	2	Included
D	-	-	3	3	2	1	Excluded
E	-	Euk	2	1	3	0	Excluded
F	Prok	Prok	3	3	2	1	Excluded

Figure S13. The schematic explanation of how eukaryotic contigs are selected based on MyCC binning, Centrifuge, and PLAST results. a) –c) represent the plots of cluster maps generated by MyCC based on marker genes, k-mer usage and depth of coverage for each k-mer (marked under the box), d) represents an example of the decision-making table for including or excluding of the contigs. The geometric shapes (triangles, squares, and circles) represent contigs in different clusters. Contigs with a hit to eukaryotes by Centrifuge or PLAST are shaded in gray. Digital numbers in each plot represent the cluster ID. The potential eukaryotic clusters are highlighted yellow. The alphabet letters A – E represent the contigs that appeared at least once in the potential eukaryotic clusters. To be included in a eukaryotic genome, a contig must appear at least twice in the potential eukaryotic clusters across different k-mer values (e.g., Contigs A-C, below). Note that 5&6mer represents a combination of 5-mer and 6-mer.

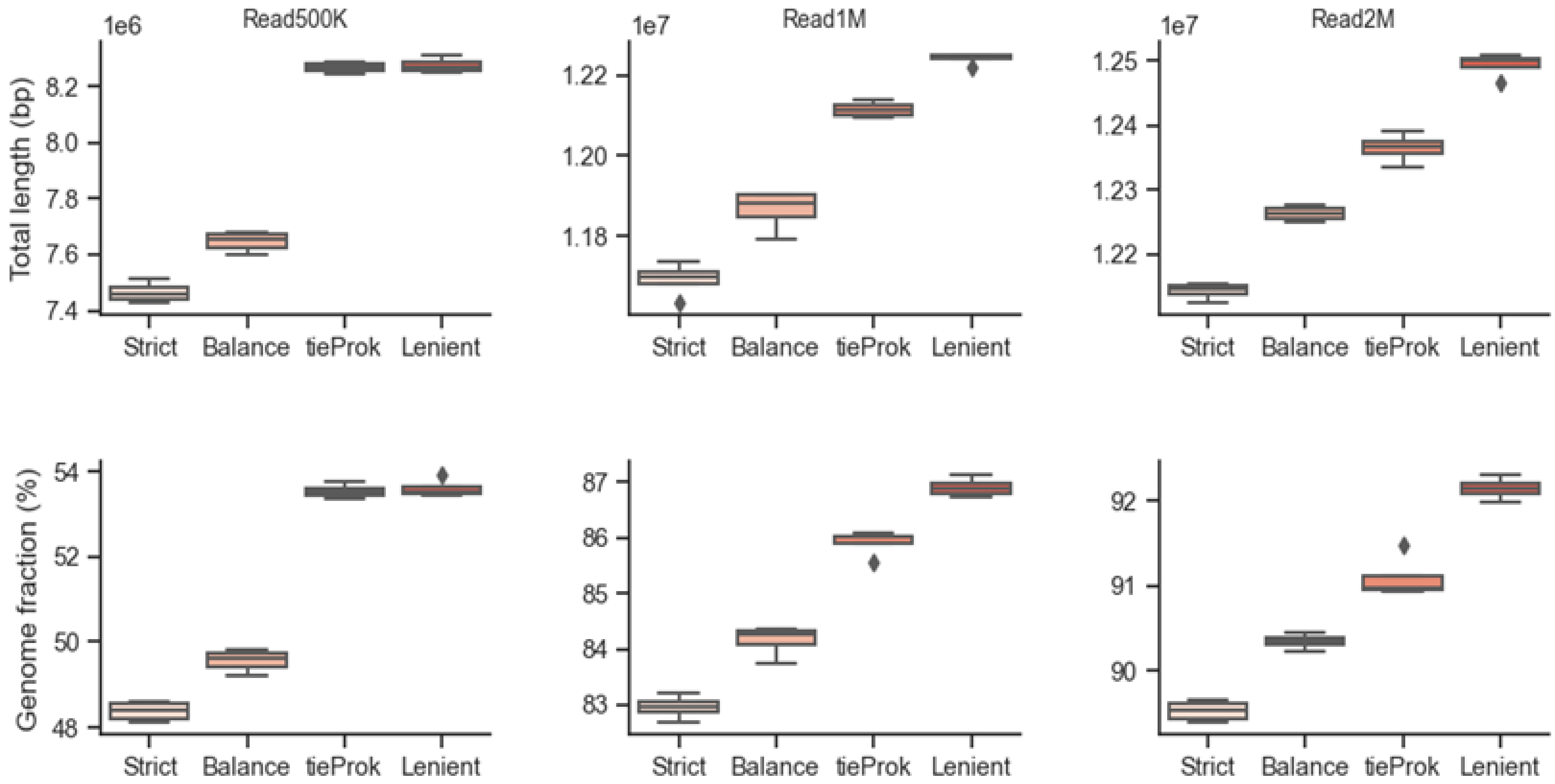

Figure S14. Plots of the effects of different parameters for EukRep in the recovery of *Blastocystis* ST1 total length (bp) and genome fraction (%) over three different *Blastocystis* read count mock community datasets (500K, 1M, and 2M) to identify optimal run parameters. EukRep algorithm stringency cut-off modes were examined (strict, balanced, and lenient) as well as the "tie" parameter, which resolves how ties are placed, eukaryote (default) or prokaryote.

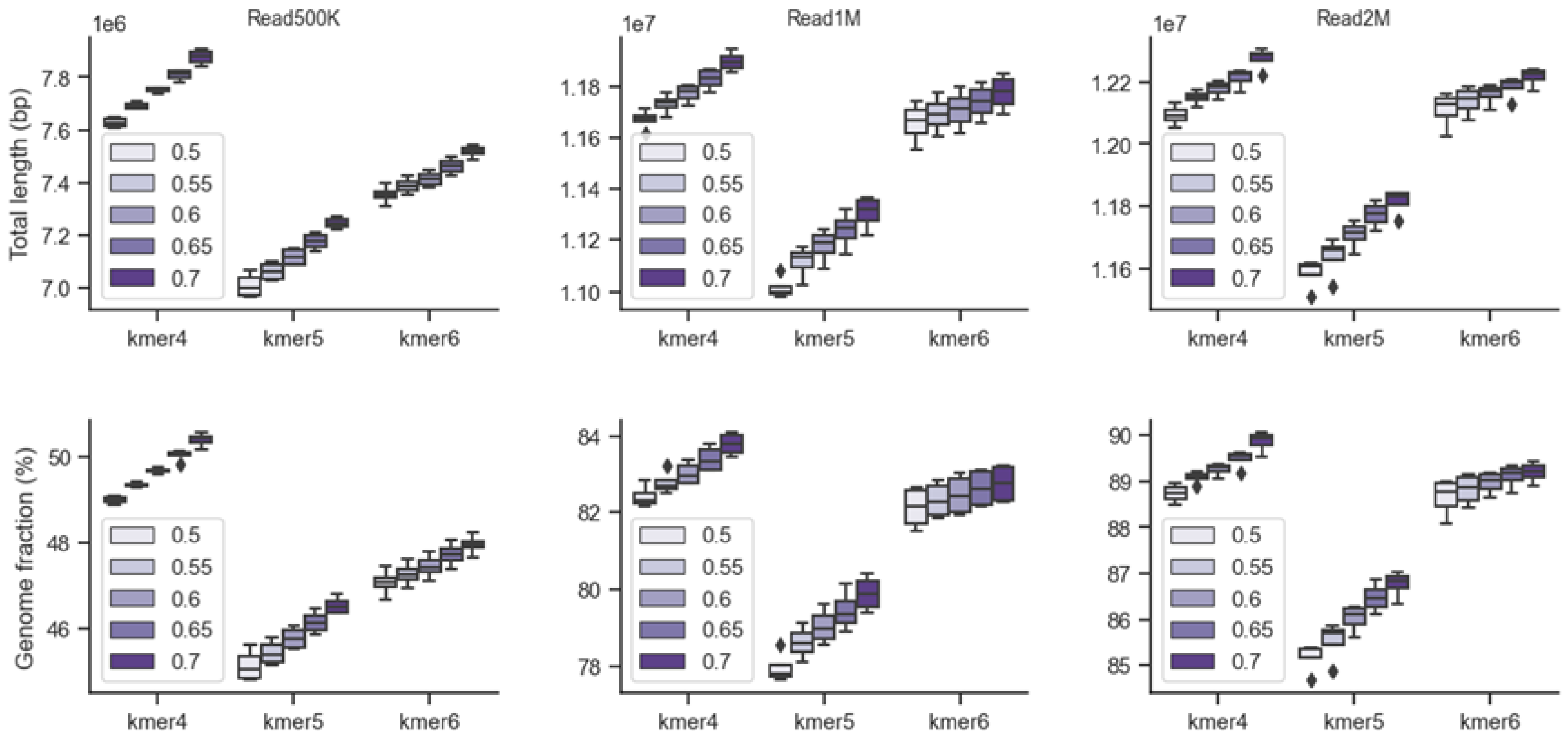

Figure S15. Plots of the effects of different parameters for Tiara in the recovery of *Blastocystis* ST1 total length (bp) and genome fraction (%) over three different *Blastocystis* read count mock community datasets (500K, 1M, and 2M) to identify optimal run parameters. Tiara k-mer sizes (4-mer, 5-mer, 6-mer) and probability thresholds (p=0.5, 0.55, 0.6, 0.65, 0.7) were examined.

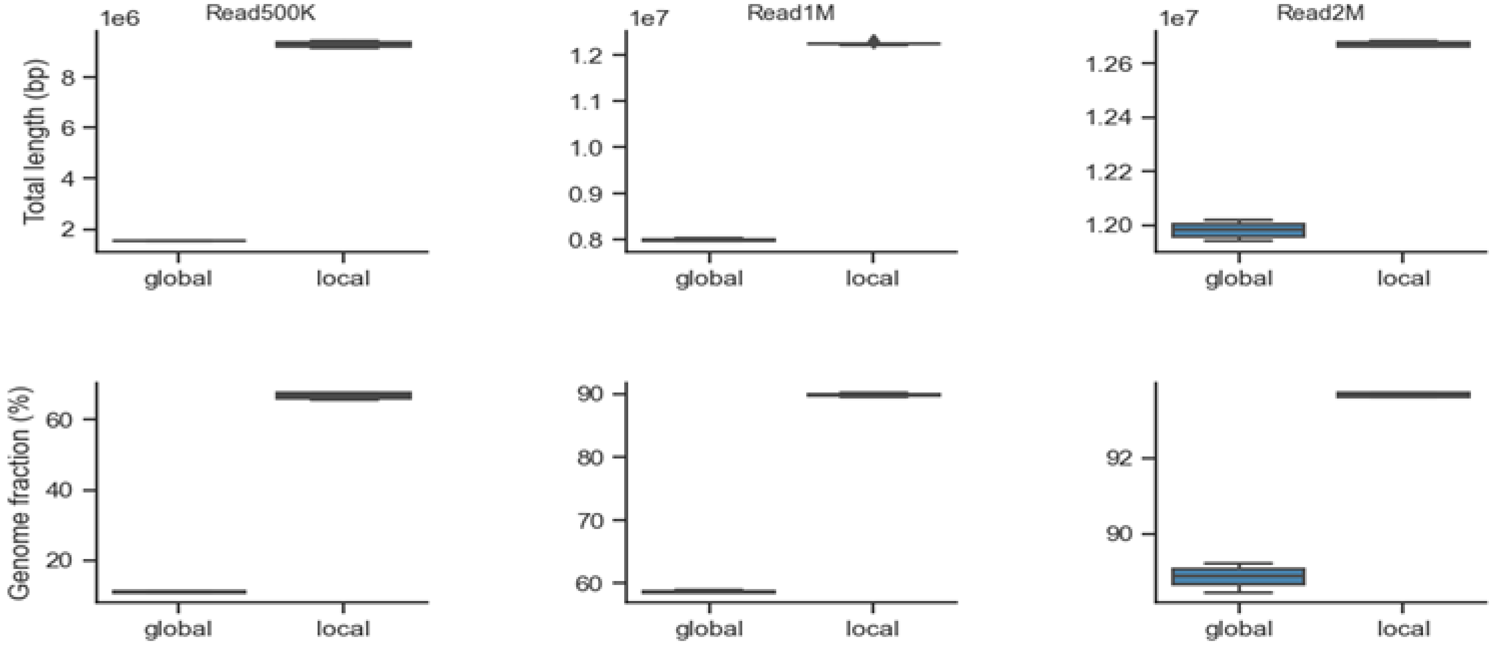

Figure S16. Plots of the effects of different parameters for Refmapping in the recovery of *Blastocystis* ST1 total length (bp) and genome fraction (%) over three different *Blastocystis* read count mock community datasets (500K, 1M, and 2M) to identify optimal run parameters. Refmapping global and local alignment for reference mapping were examined.

SUPPLEMENTARY TABLES

TABLE S1. Genomic features of published *Blastocystis* reference genomes (published before Feb 2022). BUSCO results based on stramenopile_odb10: C – Complete, F – Fragmented, M - Missing

Blastocystis subtype & isolate	GenBank Accession Number	Size (Mb)	Scaffolds	GC content (%)	Assembly Level	BUSCO Stramenopile (Total: 100 SCGs)	Year published	Reference
ST1 Nand II	GCA_001651215	16.4683	580	53.00	Scaffold	C: 100 F:0 M: 0	2017	11
ST2 Flemming	GCA_000963365	12.6931	969	54.00	Scaffold	C: 92 F:0 M: 8	2015	15
ST3 ZGR	GCA_000963385	11.6514	917	52.00	Scaffold	C: 88 F:3 M: 9	2015	15
ST4 WR1	GCA_000743755	12.9194	1301	39.70	Scaffold	C: 99 F:1 M: 0	2015	16
ST4 BT1	GCA_000963395	11.5409	849	39.90	Scaffold	C: 94 F:1 M: 5	2015	15
ST6 SSI:754	GCA_000963415	15.4178	879	43.10	Scaffold	C: 97 F:1 M: 2	2015	15
ST7 isolate B	GCA_000151665	18.8172	54	45.30	Scaffold	C: 93 F:6 M: 1	2011	17
ST7 ASY-1	GCA_003575125	10.4299	10257	52.00	Contig	C: 40 F: 20 M: 40	2018	NCBI PRJNA482946
ST8 Dmp/08-128	GCA_000963455	12.2390	947	39.70	Scaffold	C: 91 F:3 M: 6	2015	15
ST9 F5323	GCA_000963465	11.7149	871	43.00	Scaffold	C: 96 F:0 M: 4	2015	15

TABLE S2. Numbers of randomly selected *Blastocystis* ST1 Illumina sequencing reads that were combined with the synthetic ‘Mix-51-staggered’ human gut bacterial data of Miller et al (2019, SRR8304765) to create the mock metagenomic datasets used in this study.

**Identifier (# Reads)**	**Number of *Blastocystis* reads**	**Total Number of Reads (M)**	**Fraction of total dataset (%)**
300K	300,000	16.9	1.78
400K	400, 000	17	2.35
500K	500, 000	17.1	2.92
750K	750, 000	17.35	4.32
1M	1, 000, 000	17.6	5.68
2M	2, 000, 000	18.6	10.75
5M	5, 000, 000	21.6	23.15
10M	10, 000, 000	26.6	37.59

TABLE S3. Pairwise Student's t-test comparisons of total recovered length and genome fraction of the simulated mock metagenome communities using Eukfinder_longer and Eukrep (# Reads: numbers of randomly selected *Blastocystis* ST1 Illumina sequencing reads, 300K to 10M). Bonferroni correction for multiple tests was applied. The null hypothesis was that the mean total length or genome fraction of Eukfinder_long was ≤ Eukrep and the alternative was that the mean total length or genome fraction of Eukfinder_long was > Eukrep. Significant values are shown in bold.

**# Reads**	**Total Length (bp)**			**Genome Fraction (%)**		
	**t_stat**	**p_value**	**Bonferroni corrected p-value**	**t_stat**	**p_value**	**Bonferroni corrected p-value**
300K	-39.9892	**1.7204e-05**	**1.3763e-04**	-41.7586	**1.5111e-05**	**1.2089e-04**
400K	-46.5045	**1.0945e-05**	**8.7664e-05**	-56.3010	**6.1716e-06**	**4.9373e-05**
500K	-34.3827	**2.7046e-05**	**2.1637e-04**	-33.4877	**2.9268e-05**	**2.3414e-04**
750K	-19.0395	**1.5819e-04**	**1.27e-03**	-18.0790	**1.8457e-04**	**1.4765e-03**
1M	-59.4584	**5.2403e-06**	**4.1923e-05**	-53.6574	**7.1287e-06**	**5.7029e-05**
2M	-18.6709	**1.6715e-04**	**1.3372e-03**	-20.3245	**1.3020e-04**	**1.0416e-03**
5M	-20.7233	**1.2387e-04**	**9.8294e-04**	-20.2372	**1.3188e-04**	**1.0551e-03**
10M	-18.3494	**1.7658e-04**	**1.4127e-03**	-17.1189	**2.1712e-04**	**1.7370e-03**

TABLE S4. Pairwise Student's t-test comparisons of total recovered length and genome fraction of the simulated mock metagenome communities using Eukfinder_longer and Tiara (# Reads: numbers of randomly selected *Blastocystis* ST1 Illumina sequencing reads, 300K to 10M). Bonferroni correction for multiple tests was applied. The null hypothesis tested was that mean total length or genome fraction of Eukfinder_long was ≤ Tiara and the alternative was that mean total length or genome fraction of Eukfinder_long was > Tiara. Significant values are shown in bold.

**# Reads**	**Total Length (bp)**			**Genome Fraction (%)**		
	**t_stat**	**p_value**	**Bonferroni corrected p-value**	**t_stat**	**p_value**	**Bonferroni corrected p-value**
300K	-41.8166	**1.5049e-05**	**1.2039e-04**	-38.5178	**1.9249e-05**	**1.5399e-04**
400K	-128.1155	**5.2425e-07**	**4.1940e-06**	-142.4312	**3.8155e-07**	**3.0524e-06**
500K	-113.7951	**7.4808e-07**	**5.9847e-06**	-93.8298	**1.3343e-06**	**1.0674e-05**
750K	-113.7936	**7.4811e-07**	**5.9849e-06**	-106.0278	**9.2479e-07**	**7.3983e-06**
1M	-54.4037	**6.8395e-06**	**5.4716e-05**	-57.0047	**5.9460e-06**	**4.7568e-05**
2M	-35.9099	**2.3749e-05**	**1.8997e-04**	-35.2748	**2.5049e-05**	**2.0039e-04**
5M	-32.8200	**3.1087e-05**	**2.4869e-04**	-33.4750	**2.9301e-05**	**2.3441e-04**
10M	-68.8664	**3.3736e-06**	**2.6989e-05**	-60.7119	**4.9226e-06**	**3.9381e-05**

TABLE S5. Pairwise Student's t-test comparisons of total recovered length and genome fraction of the simulated mock metagenome communities (# Reads: numbers of randomly selected *Blastocystis* ST1 Illumina sequencing reads, 300K to 10M) using Eukfinder_short and Refmapping (with *Blastocystis* ST1 reference genome). Bonferroni correction for multiple tests was applied. The null hypothesis tested was that the mean total length or genome fraction of Eukfinder_short was ≤ Refmapping and the alternative was that the mean total length or genome fraction of Eukfinder_short was > Refmapping. Significant values are shown in bold.

# Reads	Total Length (bp)			Genome Fraction (%)		
	t_stat	p_value	Bonferroni corrected p-value	t_stat	p_value	Bonferroni corrected p-value
300K	97.3816	1.0	1.0	119.4961	1.0	1.0
400K	87.1572	1.0	1.0	122.9708	1.0	1.0
500K	13.4423	0.9996	1.0	22.3513	0.9999	1.0
750K	9.3843	0.9987	1.0	18.9547	0.9998	1.0
1M	4.6012	0.9904	1.0	11.0349	0.9992	1.0
2M	-12.1770	**5.9617e-04**	**0.0048**	-19.3644	**1.5041e-04**	**1.2033e-03**
5M	-17.6170	**1.9936e-04**	**0.0016**	-46.7697	**1.0761e-05**	**8.6084e-05**
10M	-40.7630	**1.6244e-05**	**1.2995e-04**	-36.7295	**2.2194e-05**	**1.7755e-04**

TABLE S6. General description of the tested human gut metagenome datasets. Samples with 3 in the name contain *Blastocystis* ST3 and those with 4 in the name contained *Blastocystis* ST4.

**Name**	**Dataset**	**# of Bases (Gbp)**	**Number of Total Reads (M)**	**Assembly (Mb)**	***Blastocystis* subtype**	***Blastocystis* Reads (M)**	**Percentage *Blastocystis* Reads to total dataset**	**Reference**
3A	SRR061218	6.0	30.2	141.0	ST3	0.50	1.67%	18
3B	SRR060363	7.7	37.9	113.4	ST3	0.48	1.27%	18
3C	ERR636351	11.1	56.7	285.7	ST3	0.42	0.74%	19
3D	ERR636414	23.3	119.7	319.4	ST3	0.82	0.68%	19
4A	ERR321632	8.5	47.2	240.9	ST4	0.45	0.95%	18
4B	ERR321638	8.7	48.4	201.0	ST4	0.53	1.09%	18
4C	ERR636359	16.5	84.2	398.7	ST4	0.59	0.70%	19
4D	ERR636397	13.6	75.3	294.1	ST4	2.63	3.4%	19

TABLE S7. General description of the recovered *Dientamoeba* genomes from human gut metagenome 4D dataset. BUSCO results based on stramenopile odb10: C – Complete, F – Fragmented, M – Missing, n – Total number of single copied genes.

**Method**	**Genome Size (Mbp)**	**# Contigs**	**BUSCO Results (eukaryota_odb10)**
**Eukfinder_short**	5.35	2190	C:2.0%, F:0.8%, M:97.2%, n:255
**Eukfinder_long**	4.89	1700	C:2.0%, F:0.8%, M:97.2%, n:255
**EukRep**	4.89	1687	C:2.0%, F:0.8%, M:97.2%, n:255
**Tiara**	2.76	1053	C:0.4%, F:1.2%, M:98.4%, n:255

TABLE S8. Number of contigs from the Tara Oceans Mediterranean Sea sample (ERR868402) identified as Eukaryota by Eukfinder_long using strict and lenient parameter settings, EukRep, and Tiara, along with estimates of inferred precision and recall. The contigs examined contained 53,411 contigs that could be pre-classified, 3,733 were inferred to be Eukaryota and 49,678 inferred to be Prokaryota or Virus. Precision was measured as true positives divided by true positives plus false positives and recall as true positives divided by true positives plus false negatives. For Tiara, the total number of contigs includes contigs classified as eukaryote or organelle.

**Method**	**Eukfinder_long strict Euk***	**Eukfinder_long strict EUnk****	**Eukfinder_long lenient Euk***	**Eukfinder_long lenient EUnk****	**EukRep**	**Tiara Euk***	**Tiara Euk & Unk****
# Contigs identified as Eukaryota	1198	33863	4700	14655	6359	4193	5985
True Positive Eukaryota Contigs	923	3530	1950	3032	1880	1494	1806
False Positive Eukaryota Contigs	275	30333	2750	11623	4479	2699	4179
False Negative Eukaryota Contigs	2810	203	1783	701	1853	2239	1927
Eukaryota Contig Precision	0.77	0.10	0.41	0.21	0.30	0.36	0.30
Eukaryota Contig Recall	0.25	0.95	0.52	0.81	0.50	0.40	0.48

*Only the contigs in the Euk group were treated as True Positive.

**Contigs in the Eukaryota or Unknown group were treated as True Positive.

TABLE S9. Parameters of Eukfinder used in this paper. Specific information corresponding for each flag is provided after the hashtag.

(1) read_prep submenu<br><br>--hcrop 10 # head trim in Trimmomatic<br>-l 15 # leading trim in Trimmomatic<br>-t 15 # trail trim in Trimmomatic<br>--wsize 40 # sliding window size in Trimmomatic<br>--qscore 25 # quality score for trimming in Trimmomatic<br>--mlen 40 # minimum read length in Trimmomatic<br>--hg GCF_000001405.39_GRCh38.p13_genomic.fna # human genome for removing host reads with Bowtie2<br>--mhlen 40   # minimum hit length in Centrifuge
(2) short_seqs submenu for Mock Community Test, Human Gut Metagenome samples<br><br>-e 0.01   # E_VALUE, threshold for plast searches<br>--pid 70   # percentage identity for plast searches<br>--cov 30   # percentage coverage for plast searches<br>--mhlen 100   # minimum hit length in Centrifuge
(3) long_seqs submenu for Mock Community Test, Human Gut Metagenome samples<br><br>-e 0.01 # E_VALUE, threshold for plast searches<br>--pid 70 # percentage identity for plast searches<br>--cov 30 # percentage coverage for plast searches<br>--mhlen 100   # minimum hit length in Centrifuge
(4) long_seqs submenu for Tara Ocean sample strict parameters<br><br>-e 0.01 # E_VALUE, threshold for plast searches<br>--pid 70 # percentage identity for plast searches<br>--cov 20 # percentage coverage for plast searches<br>--mhlen 40   # minimum hit length in Centrifuge
(5) long_seqs submenu for Tara Ocean sample lenient parameters<br><br>-e 0.01 # E_VALUE, threshold for plast searches<br>--pid 50 # percentage identity for plast searches<br>--cov 10 # percentage coverage for plast searches<br>--mhlen 25   # minimum hit length in Centrifuge

TABLE S10. Time usage, in minutes and seconds, for each method to identify potential eukaryotic reads for genome assembly. Data shown is for Human sample 3A (see Table S6) which consisted of 30.2 million reads, assembly size, 141 Mb with 30086 contigs (length ≥1000bp). Computations were performed on the Digital Research Alliance of Canada Niagra resource using 10 CPUs of a Lenovo SD350 cluster, equipped with Intel "Skylake" 2.4 GHz cores, with at least 4 GiB RAM per core. All methods were allocated 10 CPUs, with the exception of EukRep, which can only utilize a single CPU. As the supervised binning step was the same for all methods it is not included in this time assessment.

**Method**	**Total Time**	**Assembling by metaSPAdes**	**Read/Contigs Classification**[a]
Refmapping	11m 28s	1m 34s[b]	9m 54s
Eukfinder_Short	557m 40s	11m 30s[c]	546m 10s[d]
Eukfinder_Long	98m 26s	81m 20s	17m 6s[e]
EukRep	85m 28s	81m 20s	4m 8s
Tiara	82m 28s	81m 20s	1m 8s

[a] In addition to classification time, also includes time for processing and parsing results.

[b] Occurs only on reads selected after reference mapping.

[c] Occurs only on reads selected as eukaryote and unknown after the first round of Centrifuge and PLAST.

[d] Includes two rounds of Centrifuge and PLAST. One occurs before assembly with metaSPAdes and one after. Time usage for first round of classification: Centrifuge (14m 49s), PLAST (418m19s). Time usage for second round of classification: Centrifuge (2m15s), PLAST (37m40s).

[e] Includes one round of Centrifuge (2m19s) and PLAST (14m22s).

TABLE S11. Numbers of genomes present in the specialized databases by taxonomic group.

**Group**	# genomes in centrifuge DB "DB1"	# genomes in PLAST DB "DB2"
Archaea	3,662	244
Bacteria	13,623	576
Eukaryotes	1629	80
EukPathDB	243	63
Mitochondria	10,813	10,660
Virus	6,137	12,251
**Total**	**36,107**	**23,874**

TABLE S12. Contigs removed from *Blastocystis* reference genomes. MRO genomes are labelled with asterisks. Contigs matching over 50% of their total length to bacterial, archaeal, or viral sequences in the nt database and had a nucleotide identity of 80% or higher were classified as contaminants and removed from the draft genomes used to create the specialized databases.

***Blastocystis* Subtype**	**Contig**	***Blastocystis* Subtype**	**Contig**
ST2	JZRJ01000088 *	ST6	JZRM01000240
ST2	JZRJ01000923	ST6	JZRM01000270
ST3	JZRK01000047 *	ST6	JZRM01000285
ST3	JZRK010000726	ST6	JZRM01000317
ST3	JZRK01000382	ST6	JZRM01000353
ST3	JZRK01000623	ST6	JZRM01000399
ST3	JZRK01000726	ST6	JZRM01000403
ST3	JZRK01000754	ST6	JZRM01000411
ST3	JZRK01000820	ST6	JZRM01000419
ST3	JZRK01000826	ST6	JZRM01000431
ST3	JZRK01000839	ST6	JZRM01000456
ST6	JZRM01000006	ST6	JZRM01000464
ST6	JZRM01000011	ST6	JZRM01000480
ST6	JZRM01000013	ST6	JZRM01000507
ST6	JZRM01000016	ST6	JZRM01000535
ST6	JZRM01000019	ST6	JZRM01000542
ST6	JZRM01000022	ST6	JZRM01000570
ST6	JZRM01000023	ST6	JZRM01000598
ST6	JZRM01000027	ST6	JZRM01000617
ST6	JZRM01000029	ST6	JZRM01000659
ST6	JZRM01000030	ST6	JZRM01000755
ST6	JZRM01000035 *	ST6	JZRM01000790
ST6	JZRM01000036	ST6	JZRM01000826

ST6	JZRM01000052	ST6	JZRM01000830
ST6	JZRM01000058	ST6	JZRM01000879
ST6	JZRM01000081	ST8	JZRN01000022 *
ST6	JZRM01000084	ST8	JZRN01000233
ST6	JZRM01000095	ST8	JZRN01000747
ST6	JZRM01000101	ST8	JZRN01000879
ST6	JZRM01000108	ST9	JZRO01000015 *
ST6	JZRM01000117	ST9	JZRO01000142
ST6	JZRM01000122	ST9	JZRO01000228
ST6	JZRM01000123	ST9	JZRO01000234
ST6	JZRM01000137	ST9	JZRO01000235
ST6	JZRM01000150	ST9	JZRO01000417
ST6	JZRM01000163	ST9	JZRO01000444
ST6	JZRM01000189	ST9	JZRO01000788
ST6	JZRM01000198	ST9	JZRO01000789
ST6	JZRM01000214	ST9	JZRO01000859
ST6	JZRM01000230	ST9	JZRO01000871

SUPPLEMENTARY REFERENCES

1. Kim D, Song L, Breitwieser FP, Salzberg SL. 2016. Centrifuge: rapid and sensitive classification of metagenomic sequences. Genome Res 26:1721–1729
2. De Filippis F, Pasolli E, Tett A, Tarallo S, Naccarati A, De Angelis M, Neviani E, Cocolin L, Gobbetti M, Segata N, Ercolini D. 2019. Distinct Genetic and Functional Traits of Human Intestinal Prevotella copri Strains Are Associated with Different Habitual Diets. Cell Host Microbe 25:444-453.e3.
3. Stewart RD, Auffret MD, Warr A, Wiser AH, Press MO, Langford KW, Liachko I, Snelling TJ, Dewhurst RJ, Walker AW, Roehe R, Watson M. 2018 Assembly of 913 microbial genomes from metagenomic sequencing of the cow rumen. Nat Commun 9:870.
4. Chaumeil PA, Mussig AJ, Hugenholtz P, Parks DH. 2019. GTDB-Tk: a toolkit to classify genomes with the Genome Taxonomy Database. Bioinformatics 36:1925-1927.
5. Menardo F, Loiseau C, Brites D, Coscolla M, Gygli SM, Rutaihwa LK, Trauner A, Beisel C, Borrell S, Gagneux S. 2018. Treemmer: A tool to reduce large phylogenetic datasets with minimal loss of diversity. BMC Bioinformatics 19:1-8.
6. Lin H-H, Liao Y-C. 2016. Accurate binning of metagenomic contigs via automated clustering sequences using information of genomic signatures and marker genes. Sci Rep 6:24175.
7. Warrenfeltz S, Basenko EY, Crouch K, Harb OS, Kissinger JC, Roos DS, Shanmugasundram A, Silva-Franco F. 2018. EuPathDB: The Eukaryotic Pathogen Genomics Database Resource. Methods Mol Biol 1757:69-113.
8. Nguyen VH, Lavenier D. 2009. PLAST: Parallel local alignment search tool for database comparison. BMC Bioinformatics 10:1-13.
9. Altschul SF, Gish W, Miller W, Myers EW, Lipman DJ. 1990. Basic local alignment search tool. J Mol Biol 215:403-410.
10. Gentekaki E, Curtis BA, Stairs CW, Klimeš V, Eliáš M, Salas-Leiva DE, Herman EK, Eme L, Arias MC, Henrissat B, Hilliou F, Klute MJ, Suga H, Malik SB, Pightling AW, Kolisko M, Rachubinski RA, Schlacht A, Soanes DM, Tsaousis AD, Archibald JM, Ball SG, Dacks JB, Clark CG, van der Giezen M, Roger AJ. 2017. Extreme genome diversity in the hyper-prevalent parasitic eukaryote *Blastocystis*. PLoS biology 15(9):e2003769.
11. Bengtsson-Palme J, Hartmann M, Eriksson KM, Pal C, Thorell K, Larsson DGJ, Nilsson RH. 2015. METAXA2: Improved identification and taxonomic classification of small and large subunit rRNA in metagenomic data. Mol Ecol Resour 15:1403–1414.
12. Wood DE, Lu J, Langmead B. 2019. Improved metagenomic analysis with Kraken 2. Genome Biol 20:1–13.
13. West PT, Probst AJ, Grigoriev IV, Thomas BC, Banfield JF. 2018. Genome-reconstruction for eukaryotes from complex natural microbial communities. Genome Res 28:569-580.
14. Karlicki M, Antonowicz S, Karnkowska A, 2022. Tiara: deep learning-based classification

system for eukaryotic sequences. Bioinformatics 38:344-350.

15. Beghini F, Pasolli E, Truong TD, Putignani L, Cacciò SM, Segata N. 2017. Large-scale comparative metagenomics of *Blastocystis*, a common member of the human gut microbiome. ISME J 11:2848-2863.

16. Sunagawa S, Coelho LP, Chaffron S, Kultima JR, Labadie K, Salazar G, Djahanschiri B, Zeller G, Mende DR, Alberti A, Cornejo-Castillo FM, Costea PI, Cruaud C, d'Ovidio F, Engelen S, Ferrera I, Gasol JM, Guidi L, Hildebrand F, Kokoszka F, Lepoivre C, Lima-Mendez G, Poulain J, Poulos BT, Royo-Llonch M, Sarmento H, Vieira-Silva S, Dimier C, Picheral M, Searson S, Kandels-Lewis S; Tara Oceans coordinators; Bowler C, de Vargas C, Gorsky G, Grimsley N, Hingamp P, Iudicone D, Jaillon O, Not F, Ogata H, Pesant S, Speich S, Stemmann L, Sullivan MB, Weissenbach J, Wincker P, Karsenti E, Raes J, Acinas SG, Bork P. 2015. Structure and function of the global ocean microbiome. Science 348:1261359.

17. Buchfink B, Xie C, Huson DH. 2015. Fast and sensitive protein alignment using DIAMOND. Nat Methods 12:59–60.
